# SARS-CoV-2 Nsp8 suppresses MDA5 antiviral immune responses by impairing TRIM4-mediated K63-linked polyubiquitination

**DOI:** 10.1371/journal.ppat.1011792

**Published:** 2023-11-13

**Authors:** Xiaolin Zhang, Ziwei Yang, Ting Pan, Qinqin Sun, Qingyang Chen, Pei-Hui Wang, Xiaojuan Li, Ersheng Kuang

**Affiliations:** 1 Institute of Human Virology, Zhongshan School of Medicine, Sun Yat-Sen University, Guangzhou, Guangdong, China; 2 Center for Infection and Immunity Studies, School of Medicine, Sun Yat-Sen University, Shenzhen, Guangdong, China; 3 Key Laboratory for Experimental Teratology of Ministry of Education and Advanced Medical Research Institute, Cheeloo College of Medicine, Shandong University, Jinan, Shandong, China; 4 College of Clinical Medicine, Hubei University of Chinese Medicine, Wuhan, Hubei, China; 5 Key Laboratory of Tropical Disease Control (Sun Yat-Sen University), Ministry of Education, Guangzhou, Guangdong, China; Loyola University Chicago Stritch School of Medicine, UNITED STATES

## Abstract

Melanoma differentiation-associated gene-5 (MDA5) acts as a cytoplasmic RNA sensor to detect viral dsRNA and mediates antiviral innate immune responses to infection by RNA viruses. Upon recognition of viral dsRNA, MDA5 is activated with K63-linked polyubiquitination and then triggers the recruitment of MAVS and activation of TBK1 and IKKα/β, subsequently leading to IRF3 and NF-κB phosphorylation. However, the specific E3 ubiquitin ligase for MDA5 K63-polyubiquitination has not been well characterized. Great numbers of symptomatic and severe infections of SARS-CoV-2 are spreading worldwide, and the poor efficacy of treatment with type I interferon and antiviral immune agents indicates that SARS-CoV-2 escapes from antiviral immune responses via several unknown mechanisms. Here, we report that SARS-CoV-2 nonstructural protein 8 (nsp8) acts as a suppressor of antiviral innate immune and inflammatory responses to promote infection of SARS-CoV-2. It downregulates the expression of type I interferon, IFN-stimulated genes and proinflammatory cytokines by binding to MDA5 and TRIM4 and impairing TRIM4-mediated MDA5 K63-linked polyubiquitination. Our findings reveal that nsp8 mediates innate immune evasion during SARS-CoV-2 infection and may serve as a potential target for future therapeutics for SARS-CoV-2 infectious diseases.

## Author summary

The spread of SARS-CoV-2 infection and COVID-19 has caused mass casualties worldwide, and the failure of antiviral interferon treatment suggests the existence of important mechanisms of SARS-CoV-2 innate immune evasion. It has been reported that several viral proteins of severe coronaviruses suppress antiviral immune responses; however, the knowledge of immune suppression by SARS-CoV-2 remains limited. Here, we reveal that nsp8 protein of SARS-CoV-2 directly blocks the activation of viral dsRNA sensor MDA5 by impairing TRIM4-mediated K63-linked polyubiquitination and significantly downregulates antiviral immune responses. Our findings contribute to the comprehensive understanding of the direct immune evasion of SARS-CoV-2 that nsp8 suppresses the recognition of viral dsRNA and activation of MDA5 signalosome.

## Introduction

Severe acute respiratory syndrome coronavirus 2 (SARS-CoV-2) is an emerging severe coronavirus that has caused a global outbreak of Coronavirus Disease 2019 (COVID-19). This virus has infected hundreds of millions of patients and caused millions of deaths. The number of patients and deaths are still rapidly increasing; knowledge on SARS-CoV-2 infection, pathogenesis, disease and treatment remains limited, and novel targets of therapeutics and drug development are urgently needed. After three years of urgent investment and development, several kinds of vaccines and anti-SARS-CoV-2 drugs have been successfully developed and approved under emergency provisions for the prevention and treatment of severe disease.

Analysis of clinical data from different SARS-CoV-2 patients has shown that excessive cytokine release, known as a “cytokine storm”, is closely related to disease severity [1–3]. To characterize the host immune and inflammatory responses in COVID-19 patients, genome-wide RNA-sequencing analysis was performed, which indicated that the proportion of immune cells in the blood was reduced in patients who required non-ICU admission, with lower levels of G-CSF, CXCL10/IP-10, CCL2/MCP-1 and CCL3/MIP-1A detected. In addition, some anti-inflammatory cytokines, such as IL-10 and TGF-β, were found to be induced during SARS-CoV-2 infection [4]. Accordingly, compared with ICU care patients, non-ICU care patients had lower plasma levels of cytokines, including IL-2, IL-7, IL-10, GSCF, IP10, MCP1, MIP1A and TNF. Furthermore, mild cases of COVID-19 exhibited decreased plasma levels of IL-2R and IL-6 compared with severe cases, while an excessive inflammatory response was observed in dead cases [5,6]. These studies reported a tight correlation between the release of inflammatory factors and cytokines and pathogenesis of SARS-CoV-2.

SARS-CoV-2 and other coronaviruses generate massive amounts of RNA products during their infection that are then recognized by host cytosolic RNA sensors, including retinoic acid-inducible gene I (RIG-I) and melanoma differentiation-associated gene-5 (MDA5) [7,8]. Activation of RIG-I and MDA5 triggers the formation of MAVS-dependent signalosomes that induce the expression of type I interferon (IFN), IFN-stimulated genes (ISGs) and the subsequent execution of an antiviral state within the cell [9]. Upon viral RNA stimulation and recognition, RIG-I and MDA5 undergo posttranslational modification via the covalent attachment of long polyubiquitin chains or noncovalent binding of short polyubiquitin chains that function as tethers to regulate the activation of these signalosomes [10–13]. Generally, K63 polyubiquitination activates innate immune responses mediated by RIG-I and MDA5, while K48-linked polyubiquitination results in their degradation through the proteasomes, negatively regulating immune responses [14–16]. However, the regulatory and activating mechanisms underlying these outcomes during SARS-CoV-2 infection remain poorly understood.

To escape immune elimination and to survive and then replicate, coronaviruses, including SARS-CoV and MERS-CoV, have evolved strategies to inhibit or delay IFN production and responses. SARS-CoV encodes a set of accessory proteins, several of which target the innate immune response. Its nonstructural protein 1 (nsp1) binds to the 40S ribosome to inactivate translation and induces host mRNA degradation [17,18]; nsp15 was identified as an IFN antagonist to enhance immune evasion [19,20]; nsp16, a 2′O-methyltransferase (2′O-MTase), provides a cap structure at the 5′-end of viral mRNAs to evade MDA5 detection [21–23]; furthermore, open reading frame 6 (ORF6) also reduces IFN production [24]. In addition, ORF-9b localizes to mitochondrial membranes to induce the degradation of MAVS, TRAF3 and TRAF6, severely limiting host cell IFN production [25]. In addition, MERS ORF4b is involved in evasion of the innate immune response by binding α-karyopherin proteins, leading to the inhibition of NF-κB nuclear translocation [26].

Considering the numerous existing asymptomatic infected populations, it is reasonable to suggest that SARS-CoV-2 has evolved multiple effective strategies to inhibit the antiviral immune response, which is also a challenge for preventing and treating COVID-19. Consistent with these observations, a recent study showed that nsp1 of SARS-CoV-2 shuts down host mRNA translation by binding to 40S and 80S ribosomes, effectively blocking RIG-I-dependent innate immune responses [27]. The membrane protein (M) of SARS-CoV-2 was also reported to interact with RIG-I, MDA5 and TBK1, thus inhibiting the formation of the RIG-I/MDA5 signalosome and suppressing the type I and type III interferon signaling pathways [28]. In addition, nsp12 was reported to inhibit the translocation of IRF3, thereby undermining type I IFN production [29]. However, more potential immune inhibitory mechanisms mediated by viral components of SARS-CoV-2 are yet to be explored.

We performed a systematic screening and determined that SARS-CoV-2 nsp8 is a suppressor of the type I immune response upon SARS-CoV-2 infection. It decreases type I IFN production and ISG expression, by which it interacts with the MDA5 CARDs domain and then inhibits TRIM4-mediated MDA5 K63-linked polyubiquitination, thus terminates MDA5-mediated antiviral immune and inflammatory responses and facilitate SARS-CoV-2 infection.

## Results

### Nsp8 inhibits viral RNA-related antiviral responses

**Fig 1:**
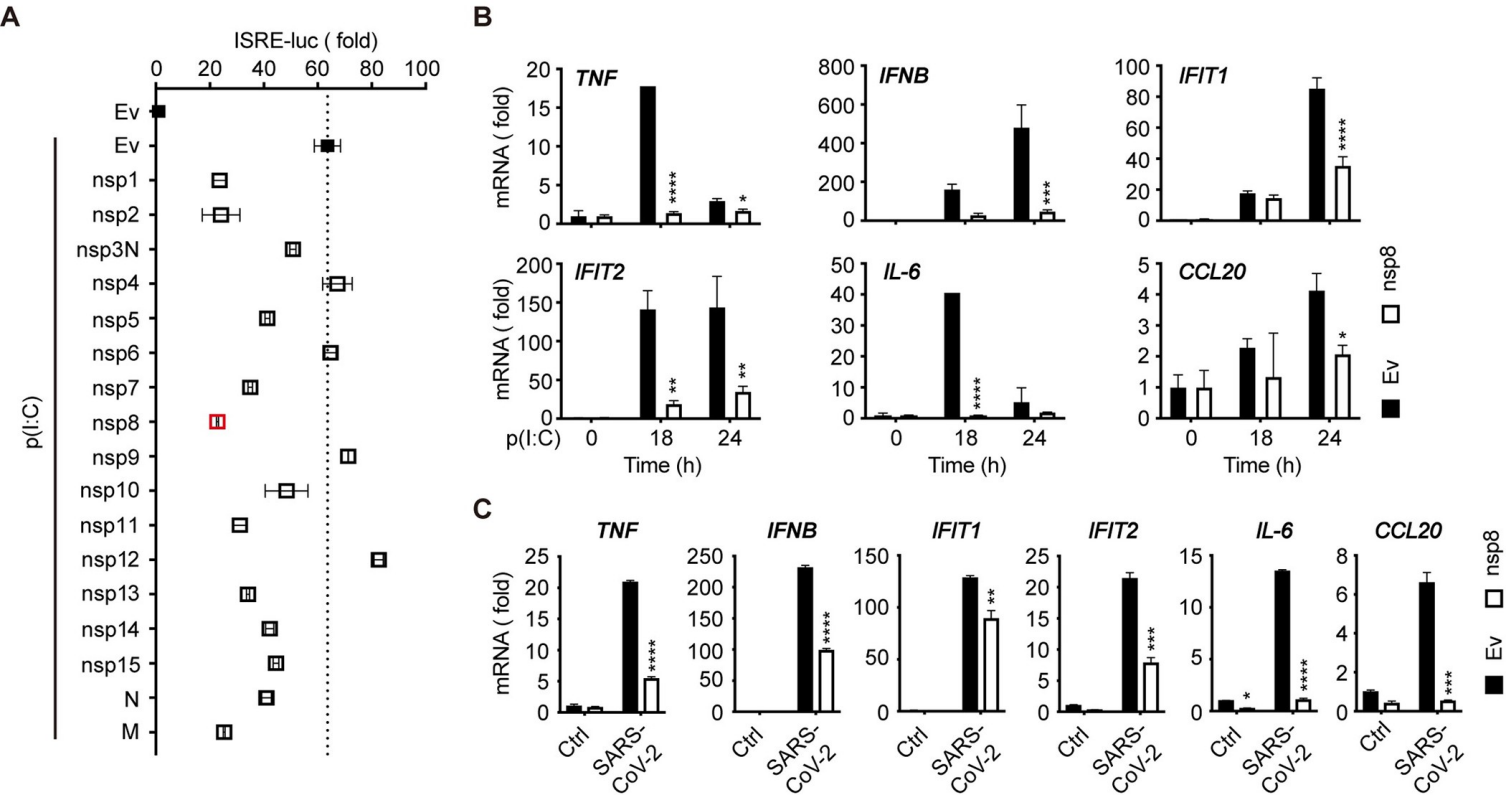
Nsp8 suppresses RNA-induced antiviral immune responses. A. HEK293T cells were transfected with a control vector (Ev) or Flag-tagged plasmids expressing 17 SARS-CoV-2 genes plus an ISRE-luc reporter plasmid. Twelve hours post transfection, cells were treated with poly(I:C) (5 μg/ml). Thirty-six hours post transfection, the cells were collected, and then cell lysates were analyzed for ISRE-luc luciferase activity. B. HEK293T cells were transfected with a control vector (Ev) or Flag-nsp8 expressing plasmid. Twenty-four hours post transfection, cells were treated with poly(I:C) (5 μg/ml) for the indicated time points and then subjected to RT-PCR analysis for *TNF*, *IFNB*, *IFIT1*, *IFIT2*, *IL-6* and *CCL20* expression. The results are shown as the mean ± SD (n = 3), *, p < 0.05; **, p < 0.01; ***, p < 0.001; ****, p < 0.0001, by Sidak’s multiple comparisons test. C. HEK293T cells were transfected with an empty vector or Flag-nsp8 along with Flag-ACE2 expressing plasmid. Twenty-four hours post transfection, cells were infected with SARS-CoV-2 (MOI = 3) for 18 h and then harvested. mRNA was extracted, reverse-transcribed and subjected to RT-PCR analysis for *TNF*, *IFNB*, *IFIT1*, *IFIT2*, *IL-6* and *CCL20* expression. The results are shown as the mean ± SD (n = 3), *, p < 0.05; **, p < 0.01; ***, p < 0.001; ****, p < 0.0001, by Sidak’s multiple comparisons test; *CCL20* by multiple t-tests.

To investigate the viral factors of SARS-CoV-2 that suppress the type I IFN signaling pathway, we screened a panel of viral nonstructural proteins (NSPs) that regulate interferon-stimulated response element (ISRE)-mediated gene expression using a luciferase reporter assay. HEK293T cells were transfected with NSP-expressing plasmids or empty vectors, followed by dsRNA analog poly(I:C) stimulation. The fold change in ISRE-luc activity showed that several viral factors, including nsp8, significantly impaired the antiviral responses of cells challenged with the dsRNA analog (Figs 1A and S1B). To further assess the attenuating effects of selected NSPs in antiviral activities, HEK293T cells were transfected with empty vectors or five viral protein-expressing plasmids that induced the greatest inhibition of ISRE-luc activity (nsp1, nsp2, nsp7, nsp8 and M) for 24 h. The cells were left untreated or challenged with the dsRNA analog poly(I:C) intracellularly for 18 h or 24 h and then harvested, and gene expression was analyzed. As shown in Figs 1B and S1A, these viral proteins, including nsp8, exhibited inhibitory effects on immune and inflammatory gene expression, and they significantly downregulated the expression of *TNF*, *IFNB*, the interferon-stimulated genes (ISGs) *IFIT1* and *IFIT2* and the proinflammatory cytokines *IL-6* and *CCL20*. Consistent with our results, nsp1 had previously been reported to inhibit the translation of type I interferon to evade the immune response, which validated our screening approach [27,30].

To confirm the attenuation of the type I IFN response by nsp8 during SARS-CoV-2 infection, nsp8-overexpressing HEK293T cells were subsequently infected with SARS-CoV-2 viral stock and both ectopic and endogenous nsp8 expression were obviously observed (S1C Fig). Consistent with the above result, the expression of interferon, ISGs and cytokines in antiviral immune and inflammatory responses was reduced robustly by nsp8 in the presence of SARS-CoV-2 infection (Fig 1C and [30]). These data demonstrated that nsp8 is an inhibitory protein of type I IFN signaling and antiviral responses during SARS-CoV-2 infection.

### Nsp8 decreases MDA5-dependent antiviral immune and inflammatory responses

**Fig 2:**
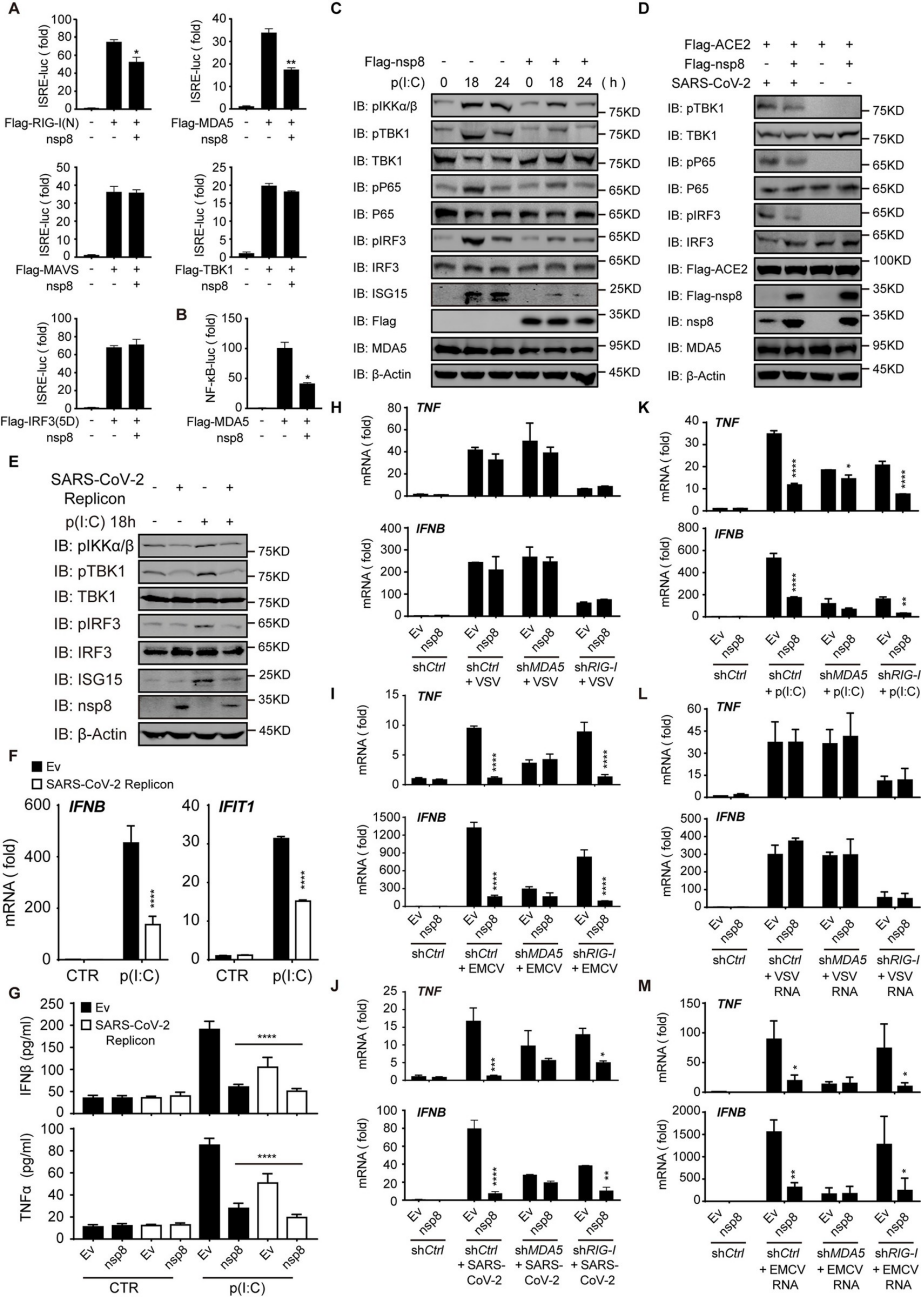
Nsp8 inhibits the MDA5-dependent immune responses. A. HEK293T cells were transfected with an empty vector or nsp8-expressing plasmid plus an ISRE-luc reporter along with RIG-I(N)-, MDA5-, MAVS-, TBK1- or IRF3(5D)-expressing plasmids respectively. Thirty-six hours post transfection, the cells were collected, and then cell lysates were analyzed for ISRE-luc activity. The results are shown as the mean ± SD (n = 3), *, p < 0.05; **, p < 0.01, by Sidak’s multiple comparisons test. B. HEK293T cells were transfected with an empty vector or nsp8-expressing plasmid plus an NF-kB-luc reporter along with MDA5-expressing plasmids. Thirty-six hours post transfection, the cells were collected, and then cell lysates were analyzed for NF-kB-luc activity. The results are shown as the mean ± SD (n = 3), *, p < 0.05, by Sidak’s multiple comparisons test. C. HEK293T cells were transfected with an empty vector or Flag-nsp8 expressing plasmid. Twenty-four hours post transfection, cells were treated with poly(I:C) (5 μg/ml) for the indicated time points and then harvested. Whole cell extracts were analyzed by western blotting as indicated. D. HEK293T cells were transfected with an empty vector or Flag-nsp8 along with Flag-ACE2 expressing plasmid. Twenty-four hours post transfection, cells were infected with SARS-CoV-2 (MOI = 3) for 18 h and then harvested. Whole cell extracts were analyzed by immunoblotting as indicated. E-F. HEK293T cells were transfected with SARS-CoV-2 replicon DNA. Twenty-four hours post transfection, cells were treated with poly(I:C) (5 μg/ml) for 18h and then harvested. Whole cell extracts were analyzed by western blotting as indicated (E), and total RNA were extracted and subjected to real-time PCR analysis (F). The results are shown as the mean ± SD (n = 3), ****, p < 0.0001, by Sidak’s multiple comparisons test. G. A549 control cells or cells harboring SARS-CoV-2 replicon were transfected with an empty vector or Flag-nsp8. After 24 h, cells were treated with poly(I:C) (5 μg/ml) for 24h, and the release of IFNβ or TNFα was measured by enzyme-linked immunosorbent assay (ELISA). The results are shown as the mean ± SD (n = 3), ****, p < 0.0001, by Sidak’s multiple comparisons test. H-J. Control (shCtrl), MDA5 knockdown (shMDA5) or RIG-I knockdown (shRIG-I) HEK293T cells were transfected with an empty vector or Flag-nsp8. Twenty-four hours post transfection, cells were infected with VSV (MOI = 1) (H) or EMCV (MOI = 0.25) (I) or SARS-CoV-2 (MOI = 3) (J) for 18 h, and then total RNA was extracted and subjected to RT-PCR analysis for *TNF*, *IFNB*. The results are shown as the mean ± SD (n = 3), *, p < 0.05; **, p < 0.01; ***, p < 0.001; ****, p < 0.0001, by Sidak’s multiple comparisons test. K-M. Control (shCtrl), MDA5 knockdown (shMDA5) or RIG-I knockdown (shRIG-I) HEK293T cells were transfected with an empty vector or Flag-nsp8 expressing plasmid. Twenty-four hours post transfection, cells were transfected with poly(I:C) (5 μg/ml) (K), VSV RNA (500 ng/ml) (L) or EMCV RNA (500 ng/ml) (M) for 24 h, and then total RNA was extracted and subjected to RT-PCR analysis for *TNF* and *IFNB* expression. The results are shown as the mean ± SD (n = 3), *, p < 0.05; **, p < 0.01; ***, p < 0.001; ****, p < 0.0001, by Sidak’s multiple comparisons test.

To further identify the target and pathway by which nsp8 attenuates antiviral immune responses, we assessed the key components associated with RIG-I-like receptors (RLRs), including MDA5, RIG-I, MAVS, TBK1 and IRF3, that regulate ISRE-luc activation in the absence or presence of nsp8 expression. The results showed that nsp8 markedly inhibited MDA5-mediated ISRE-luc activity, had a weak inhibitory effect on RIG-I and did not inhibit MAVS-, TBK1- or IRF3-induced activities (Fig 2A). The immunoblot analysis showed that nsp8 slightly impaired RIG-I and MDA5 protein levels while exhibiting no effect on MAVS, TBK1 or IRF3 expression (S2A Fig). Similarly, we did not observe that nsp8 can affect cGAS/STING-induced antiviral responses (S2B Fig). Nsp8 also exhibited the full inhibition on ISRE activity and hardly affect TLR3 expression under poly(I:C) stimulation in TLR3-depleted cells compared with control cells, indicating that TLR3 is not involved in nsp8 inhibition (S2C Fig). In addition, we also found that nsp8 suppressed MDA5-induced NF-κB-luc activity (Fig 2B), and we did not observe obvious changes in cell phenotype or cell viability in the absence or presence of nsp8 overexpression (S2D Fig). To further understand whether nsp8 inhibited both the IRF3 and NF-κB pathways, HEK293T cells were transfected with the empty vector or nsp8-expressing plasmid, followed by poly(I:C) stimulation for the indicated times. Immunoblotting analysis was used to examine the effect of nsp8 on the phosphorylation of IKKα/β, TBK1, p65 and IRF3 (Fig 2C), and we found that the phosphorylated IKKα/β and TBK1 levels were decreased in nsp8-overexpressing cells compared with control cells after poly(I:C) stimulation. As downstream transcription factors that are phosphorylated and activated by TBK1 or IKKα/β, IRF3 phosphorylation was drastically inhibited in nsp8-overexpressing cells, and NF-κB signaling was strongly inhibited by nsp8 overexpression, as indicated by the decrease in p65 phosphorylation. As a result, the expression of ISG15 was induced by poly(I:C) stimulation and was reduced in the presence of overexpressed nsp8 (Fig 2C). Importantly, the phosphorylation levels of TBK1, p65 and IRF3 were greatly induced by SARS-CoV-2 infection, while they were decreased by nsp8 overexpression during SARS-CoV-2 infection in ACE2-overexpressing HEK293T cells (Fig 2D). Under both conditions, the level of MDA5 expression was weakly affected by nsp8 overexpression. In addition, TBK1, IKKα/β and IRF3 phosphorylation and ISG15 expression were decreased in the presence of endogenous nsp8 in SARS-CoV-2-infected cells compared with those in uninfected control cells after poly(I:C) stimulation (Fig 2E). As a result, both *IFNB* and *IFIT1* expression were suppressed (Fig 2F). In addition, the poly(I:C)-induced production of IFNβ and TNFα were also suppressed by SARS-CoV-2 infection or nsp8 overexpression (Fig 2G). Taken together, these results suggest that nsp8 suppresses the activation of both IRF3 and NF-κB signaling pathways and antiviral innate immune responses.

Several studies have shown that MDA5 mainly senses SARS-CoV-2 infection to activate antiviral immune responses [8,31,32]. We hypothesized that nsp8 may preferentially regulate MDA5-mediated responses rather than RIG-I-mediated responses. To identify the potential target of nsp8, we knocked down MDA5 or RIG-I in HEK293T cells (S2G Fig), overexpressed nsp8 and stimulated the cells via infection with three kinds of RNA viruses. Analysis of *TNF* and *IFNB* expression by RT-PCR showed that *TNF* and *IFNB* production was inhibited by RIG-I depletion but not by MDA5 depletion under VSV infection and none was affected by nsp8 overexpression (Fig 2H). In contrast, MDA5 depletion but not RIG-I depletion greatly reduced these innate immune responses to EMCV infection, and nsp8-mediated inhibition of *TNF* and *IFNB* was almost completely abolished by MDA5 depletion but not by RIG-I depletion (Fig 2I). Furthermore, either MDA5 depletion or RIG-I depletion reduced *TNF* and *IFNB* expression during SARS-CoV-2 infection, and nsp8-mediated inhibition of *TNF* and *IFNB* was greatly abolished by MDA5 depletion but only slightly attenuated by RIG-I depletion in the context of SARS-CoV-2 infection (Fig 2J), emphasizing that nsp8 mainly inhibits MDA5-mediated responses during SARS-CoV-2 infection. Similarly, MDA5 or RIG-I depletion alone reduced poly(I:C)-induced *TNF* and *IFNB* expression in HEK293T cells, and MDA5 knockdown undermined nsp8-mediated suppression of the expression of *TNF* and *IFNB* compared with control treatment, while the depletion of RIG-I hardly impacted the inhibitory effect of nsp8 in the presence of poly(I:C) stimulation (Fig 2K). To further clarify the specificity of nsp8-mediated inhibition on MDA5 signaling, purified VSV RNA or EMCV RNA was used as a RIG-I-specific or MDA5-specific RNA ligand, respectively. RIG-I depletion dramatically decreased *TNF* and *IFNB* expression, while MDA5 depletion hardly affected this expression, and nsp8 exhibited no inhibitory effect in the presence of VSV RNA stimulation (Fig 2L). In the presence of EMCV RNA stimulation, either nsp8 expression or MDA5 depletion similarly reduced EMCV RNA-induced *TNF* and *IFNB* expression but RIG-I depletion did not, and nsp8-mediated inhibition of *TNF* and *IFNB* was greatly abolished by MDA5 depletion but barely affected by RIG-I depletion (Fig 2M), supporting that nsp8 suppresses MDA5-specific innate immune responses. However, nsp8-mediated inhibition of *TNF* and *IFNB* was not observed under stimulation with poly (dA: dT) or during HSV-1 infection (S2E and S2F Fig). Given these findings, nsp8 suppresses antiviral immune and inflammatory responses through the MDA5 pathway.

### Nsp8 interacts with MDA5

**Fig 3:**
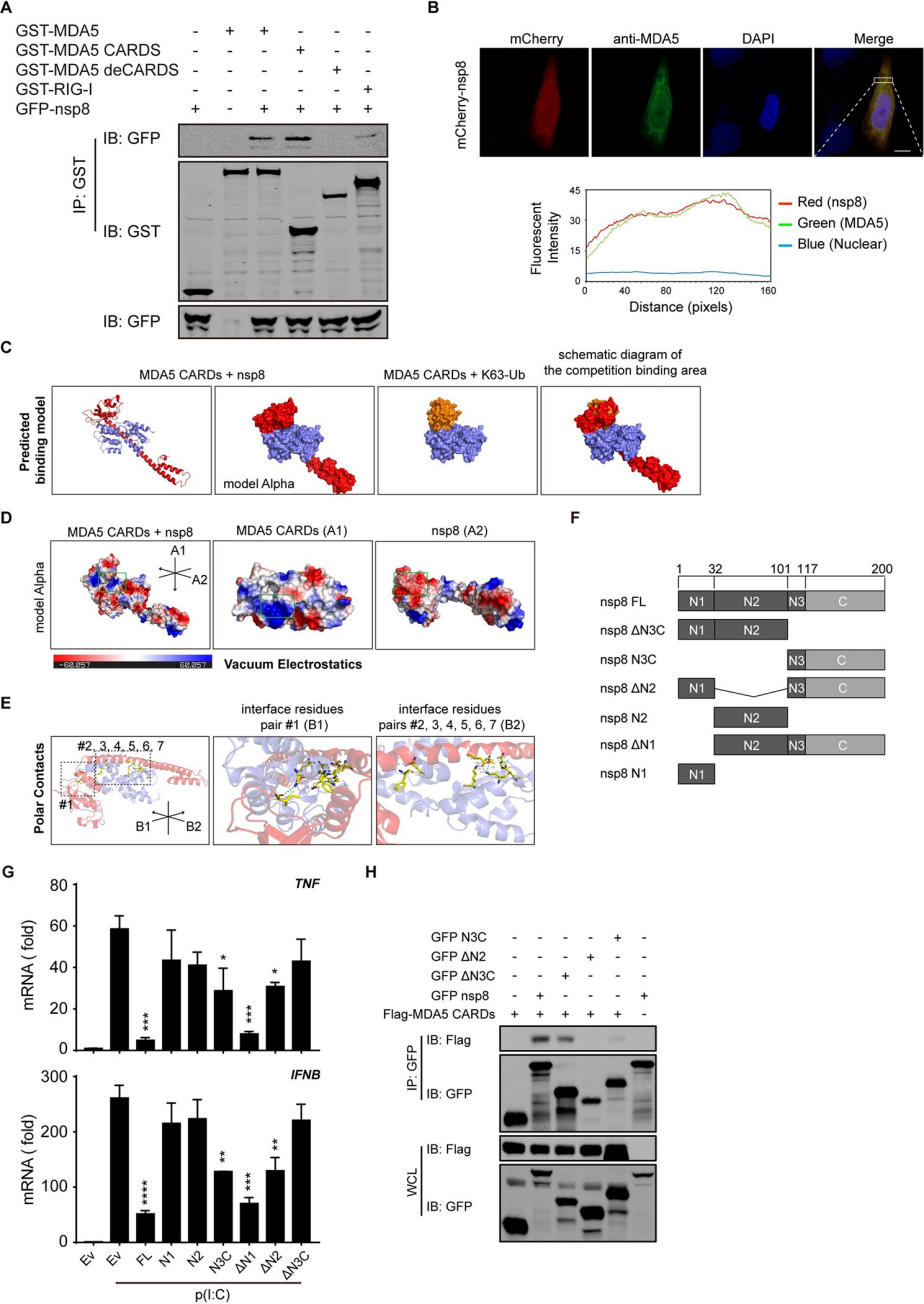
Nsp8 interacts with MDA5 at the CARDs domain. A. HEK293T cells were transfected with GFP-nsp8 and full-length GST-MDA5 or GST-MDA5 ΔCARDs or GST-MDA5 CARDs or full-length GST-RIG-I expressing plasmid, and after 36 h, the cells were collected and lysed. Then, cell lysates were subjected to coimmunoprecipitation using anti-GST beads, followed by immunoblotting with the indicated antibodies. B. HeLa cells were transfected with mCherry-nsp8. Twenty-four hours post transfection, cells were fixed and stained with anti-MDA5 antibody, and the images were visualized by confocal microscopy analysis. Scale bar: 10 μm. The coefficient of colocalization was determined by qualitative analysis of the fluorescence intensity of the selected area in Merge. C. PDB structures were input into ZDOCK Server for docking calculation separately. The predicted binding models of MDA5 CARDs with nsp8 and MDA5 CARDs with K63-Ub were processed in PyMOL for demonstration. Model alpha simulates the protein surface. Red chain, nsp8; violet chain, MDA5 CARDs; brown chain, K63-Ub. D. Model alpha in (C) was subjected to vacuum electrostatics calculation in PyMOL. A1 and A2 indicate the viewing angle in the green frame. The green frame indicates the contact area demonstrated in A1 and A2. Scale bar indicates the range of vacuum electrostatics. E. Polar contacts within interface of MDA5 CARDs-nsp8 were demonstrated with PyMOL. B1, B2 indicates the viewing angle of dashed borders. #1, 2, 3, 4, 5, 6, 7 indicates paired residues. Paired residues were highlighted with sticks model in yellow color. Green dashed lines indicate polar contacts between paired residues, number besides dashed line indicates distance between two atoms connected (Å). F. The diagram of nsp8 and its truncated mutants. N-terminal domains (N): N1, N2 and N3. C-terminal domains (C): C. G. HEK293T cells were transfected with empty vector or nsp8 truncated mutants. Twenty-four hours post transfection, cells were treated with poly(I:C) (5 μg/ml) for 18 h and then total RNA was extracted and subjected to RT-PCR analysis for *TNF* and *IFNB* expression. The results are shown as the mean ± SD (n = 3), *, p < 0.05; **, p < 0.01; ***, p < 0.001; ****, p < 0.0001, by Sidak’s multiple comparisons test. H. HEK293T cells were transfected with GFP-nsp8 and its truncated mutants along with Flag-MDA5 CARDs, and after 36 h, the cells were collected and lysed. Then, cell lysates were subjected to coimmunoprecipitation using anti-GFP beads, followed by immunoblotting with the indicated antibodies.

To investigate the mechanism by which nsp8 specifically inhibits the MDA5-mediated signaling pathway, we performed coimmunoprecipitation (co-IP) analysis and found that nsp8 interacts with MDA5 (Fig 3A). Furthermore, we mapped the binding domain and determined that its CARDs domains are responsible for the nsp8 interaction and that deletion of the CARDs domains abolished their interaction. We also detected the association between nsp8 and RIG-I, and coimmunoprecipitation analyses showed that nsp8 weakly interacts with RIG-I (Fig 3A). In addition, confocal microscopy demonstrated that nsp8 largely colocalized with endogenous MDA5 inside cells (Fig 3B). These results suggest that nsp8 interacts with MDA5 to directly suppress MDA5-mediated immune responses.

To further understand the molecular mechanism by which nsp8 interacts and interferes with MDA5, we predicted the MDA5 CARDs, nsp8 and K63-Ub tertiary structures with SWISS-MODEL, and then the predicted structures were input into ZDOCK SERVER for simulation. Predicted docking models were processed in PyMOL for visualization. Surprisingly, we found that nsp8 possesses a long α-helix (N2) (Figs 3C and S3A). The C-terminal or N-terminal domain of this predicted nsp8 structure in the nsp8-MDA5 complex was very similar to nsp8a or nsp8b isoform, respectively, one of the nsp8 structures in the viral nsp12-nsp7-nsp8 RNA polymerase complex, which carries two nsp8 molecules (S3B Fig, [33,34]). The long α-helix is tightly packed in the ravines formed by the two α-helixes of MDA5 CARDs, and the random coil and a short α-helix in the N terminus of nsp8 (N3 plus C) occupy the area or space that interacts with K63-Ub (Figs 3C and S3C). Further calculation of vacuum electrostatics for this binding model demonstrated that the contact area in the chain of MDA5 CARDs is positively charged, while the corresponding area in the chain of nsp8 is negatively charged (Figs 3D and S3D), implying that there is a likely interaction of these two structures. We further searched the polar contacts in the interface of the binding model with PyMOL and found that 7 paired residues anchor with each other, one locates in the C-terminal coil of nsp8 while the others are in the long α-helix (Fig 3E). Thus, computer-based molecular structural prediction and modeling implies that nsp8 interacts with the MDA5 CARDs domains probably through ionic interactions and dipolar surfaces between the nsp8 and MDA5 CARDs binding pockets. As shown in this binding model, an interaction between nsp8 and MDA5 CARDs was observed, while MDA5 ΔCARDs no longer bound to nsp8 (Fig 3A). To further assess this binding model using functional and biochemical experiments, we constructed a series of plasmids encoding different nsp8 variants, including α-helix domain or random coil domain deletion (ΔN3C, N3C, ΔN2, N2, ΔN1, N1) and some point mutations (MUT1, MUT2, MUT3, MUT4, MUT5 and MUT6) (Figs 3F and S3E). As expected, the long α-helix domain N2, which localized in the ravines formed by the two α-helixes of MDA5 CARDs with 6 paired residue anchorages, and the random coil domain C-sheet, which occupied the binding area of K63-Ub on MDA5 CARDs, dominated the inhibitory effect of nsp8. Deletion of C-sheets (ΔN3C) in nsp8 completely abolished the suppression of both *TNF* and *IFNB* expression, and deletion of N1-N2 (single C-sheets) or deletion of N2 alone attenuated the suppression *TNF* and *IFNB* expression. Moreover, single N1 or N2 barely suppressed *TNF* and *IFNB* expression while deletion of N1 showed the full inhibitory effect (Fig 3G). Consistent with the above results, co-IP analysis showed that the N2 domain but not the C-sheet plus N3 domain dominated the interaction with the MDA5 CARDs domain. Deletion of the C-sheet plus N3 (ΔN3C) did not abolish this interaction, while deletion of N2 did (Fig 3H). These results suggest that N2 interacts with MDA5 while N3C is responsible for inhibition. In addition, single-point mutations of the paired residues in the N2 and C-sheet domain barely counteracted the inhibitory effect of wild-type nsp8, and two-point mutations (E79A/D80A or Q90A/I122A) moderately reduced the inhibitory effect of nsp8 (S3E Fig). The loss of one of the anchoring pairs may not affect their association and inhibition, while two-point mutants of nsp8 might attenuate the interaction with MDA5 CARDs (Figs 3E and S3E). These results indicate that the long α-helix domain N2 of nsp8 interacts with the MDA5 CARDs domain, while the C-sheet generates an inhibitory effect.

To exclude the possibility that nsp8 may interfere with RIG-I in a similar manner, we predicted the binding model of RIG-I CARDs and nsp8 and K63-Ub tertiary structures with ZDOCK SERVER. Distinct from MDA5 CARDs, computational structural modeling showed that RIG-I CARDs dock with nsp8 in an incompact manner, while K63-Ub links with RIG-I CARDs at a different site (S3F Fig). Further calculation of the vacuum electrostatics of their contact area showed that the chain of nsp8 is positively charged, RIG-I CARDs also contain positively charged corresponding areas (S3G Fig), only 2 paired residues in nsp8 weakly anchor with RIG-I CARDs (S3H Fig), while 7 paired residues tightly anchor with MDA5 CARDs (Fig 3E). In addition, coimmunoprecipitation analysis showed that RIG-I weakly interacted with nsp8, validating that nsp8 did not mainly target RIG-I (Fig 3A). Therefore, these results suggest that nsp8 preferentially interacts with MDA5 but not RIG-I, even though RIG-I CARDs share a similar conformation with MDA5 CARDs.

### Nsp8 suppresses MDA5 K63-linked polyubiquitination

**Fig 4:**
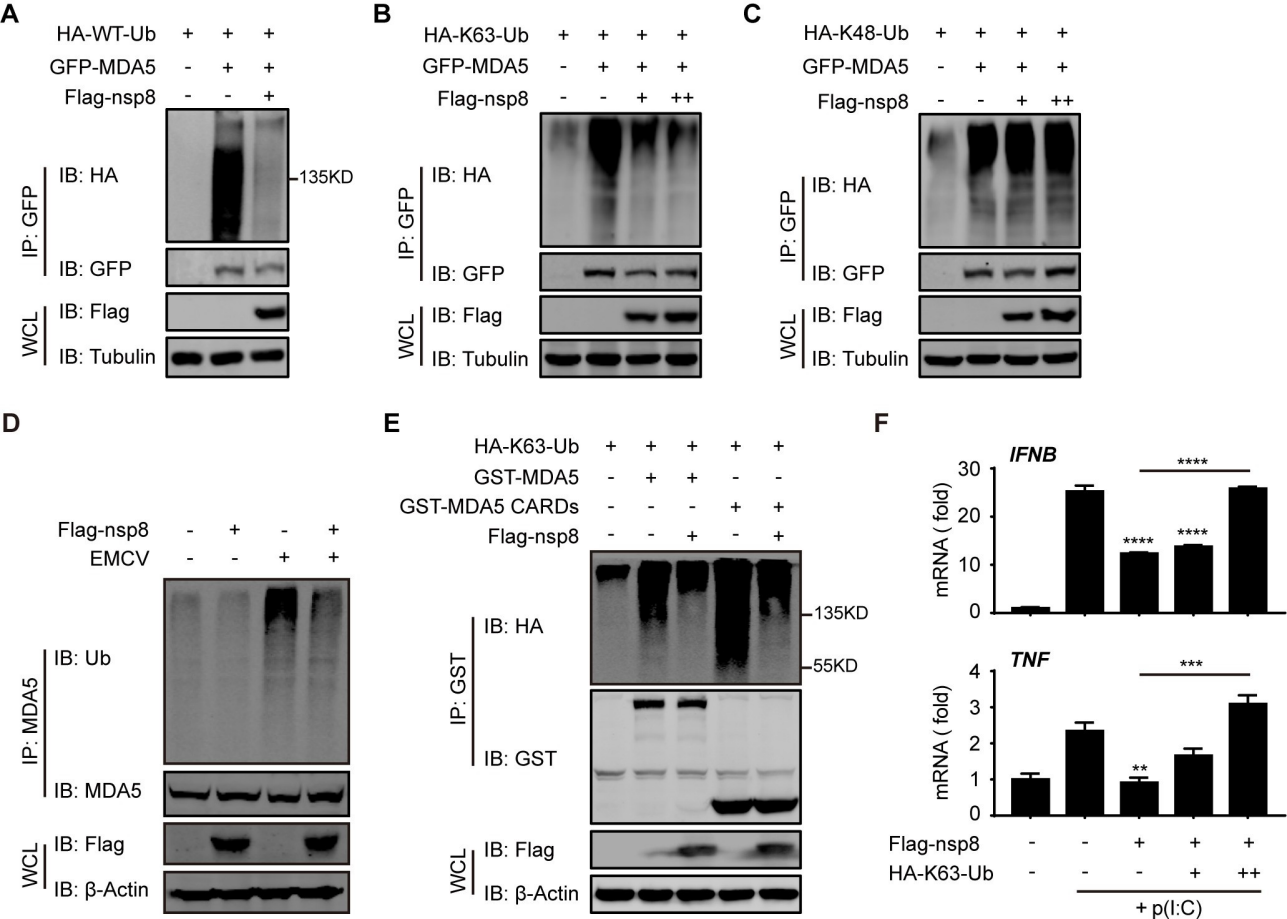
Nsp8 suppresses K63-linked polyubiquitination of MDA5. A-C. HEK293T cells were co-transfected with HA-WT-Ub (A), HA-K63-Ub (B) or HA-K48-Ub (C) and GFP-MDA5 or GFP-tagged empty vector plus Flag-nsp8. Twenty-four hours post transfection, cells were treated with MG132 (10 μM) for 4 h, and then cells were collected and lysed in 0.1% SDS-containing lysis buffer. Cell lysates were subjected to coimmunoprecipitation using anti-GFP beads, followed by immunoblotting analysis with the indicated antibodies. D. HEK293T cells were transfected with an empty vector or Flag-nsp8-expressing plasmid. Twenty-four hours post transfection, cells were infected with EMCV (MOI = 0.25) for 18 h, and then cells were collected and lysed in 0.1% SDS-containing lysis buffer. Cell lysates were subjected to coimmunoprecipitation using protein A/G beads with anti-MDA5 antibody, followed by immunoblotting analysis with the indicated antibodies. E. HEK293T cells were co-transfected with HA-K63-Ub and GST-MDA5, GST-MDA5 CARDs or GST-tagged empty vector plus Flag-nsp8. Thirty-six hours post transfection, cells were collected and lysed in 0.1% SDS-containing lysis buffer. Cell lysates were subjected to coimmunoprecipitation using anti-GST agarose beads, followed by immunoblotting analysis with the indicated antibodies. F. HEK293T cells were transfected with an empty vector or Flag-nsp8, together with increasing amounts of HA-K63-Ub. Twenty-four hours post transfection, cells were treated with poly(I:C) (5 μg/ml) for 18 h and then total RNA was extracted and subjected to RT-PCR analysis for *TNF* and *IFNB* expression. The results are shown as the mean ± SD (n = 3), **, p < 0.01; ***, p < 0.001, ****, p < 0.0001, by Sidak’s multiple comparisons test.

It is well documented that upon virus infection, the MDA5 CARDs domain undergoes K63-linked polyubiquitination and recruits MAVS to form a signalosome [13]. The structural prediction of the nsp8-MDA5 CARDs interaction showed that nsp8 may interrupt this process since it interacts with MDA5 at its CARDs domain and shields the binding area or space for K63-ubiquitin linkage (Figs 3C and S3C). Then, we sought to determine how nsp8 inhibits MDA5 activation. The polyubiquitination of MDA5 was analyzed in the presence or absence of nsp8 expression. The MDA5-expressing plasmid was co-transfected into HEK293T cells with a WT-, K63- or K48- linked ubiquitin-expressing plasmid, and a ubiquitination assay showed that MDA5 WT- and K63- linked polyubiquitination were strongly inhibited by nsp8 overexpression (Fig 4A and 4B), while K48-linked polyubiquitination was barely affected (Fig 4C). In vivo ubiquitination further revealed that nsp8 inhibited EMCV-induced endogenous MDA5 polyubiquitination (Fig 4D). As it has been reported that TRIM65 mediates the K63-linked ubiquitination of MDA5 at lysine 743 of the helicase domain [35], we performed the same experiment using MDA5 CARDs. The results showed that helicase domain-deleted MDA5 still underwent K63-linked polyubiquitination and that this polyubiquitination was still suppressed by nsp8 (Fig 4E), which excluded the possibility that nsp8 suppresses the K63-linked polyubiquitination of MDA5 on the helicase domain. In addition, we also performed polyubiquitination analysis to examine K63-linked polyubiquitination of MDA5 in the absence or presence of nsp2, nsp7 or nsp8, which all inhibited dsRNA-induced IFN-mediated antiviral responses (S1A Fig). Nsp2 or nsp7 slightly decreased the K63-linked polyubiquitination of MDA5, while nsp8 strongly did (S4A Fig). This result demonstrates that nsp8 plays a specific inhibitory role in MDA5 K63-linked polyubiquitination. Based on our binding model of nsp8 and MDA5 CARDs (Fig 3C), we hypothesized that nsp8 may compete with K63-Ub for binding or shield the linkage of K63-polyubiquitination on MDA5 CARDs upon activation. Thus, we transfected HEK293T cells with Flag-nsp8 and increasing amounts of K63-Ub overexpression. The results showed that the inhibitory effect of nsp8 on *IFNB* and *TNF* expression was gradually restored by increased K63-Ub expression (Figs 4F and S4B). Thus, these results reveal that nsp8 interferes with the MDA5 signalosome by inhibiting the K63-linked polyubiquitination of MDA5.

### Nsp8 suppresses TRIM4-mediated MDA5 K63-linked polyubiquitination and antiviral responses

**Fig 5:**
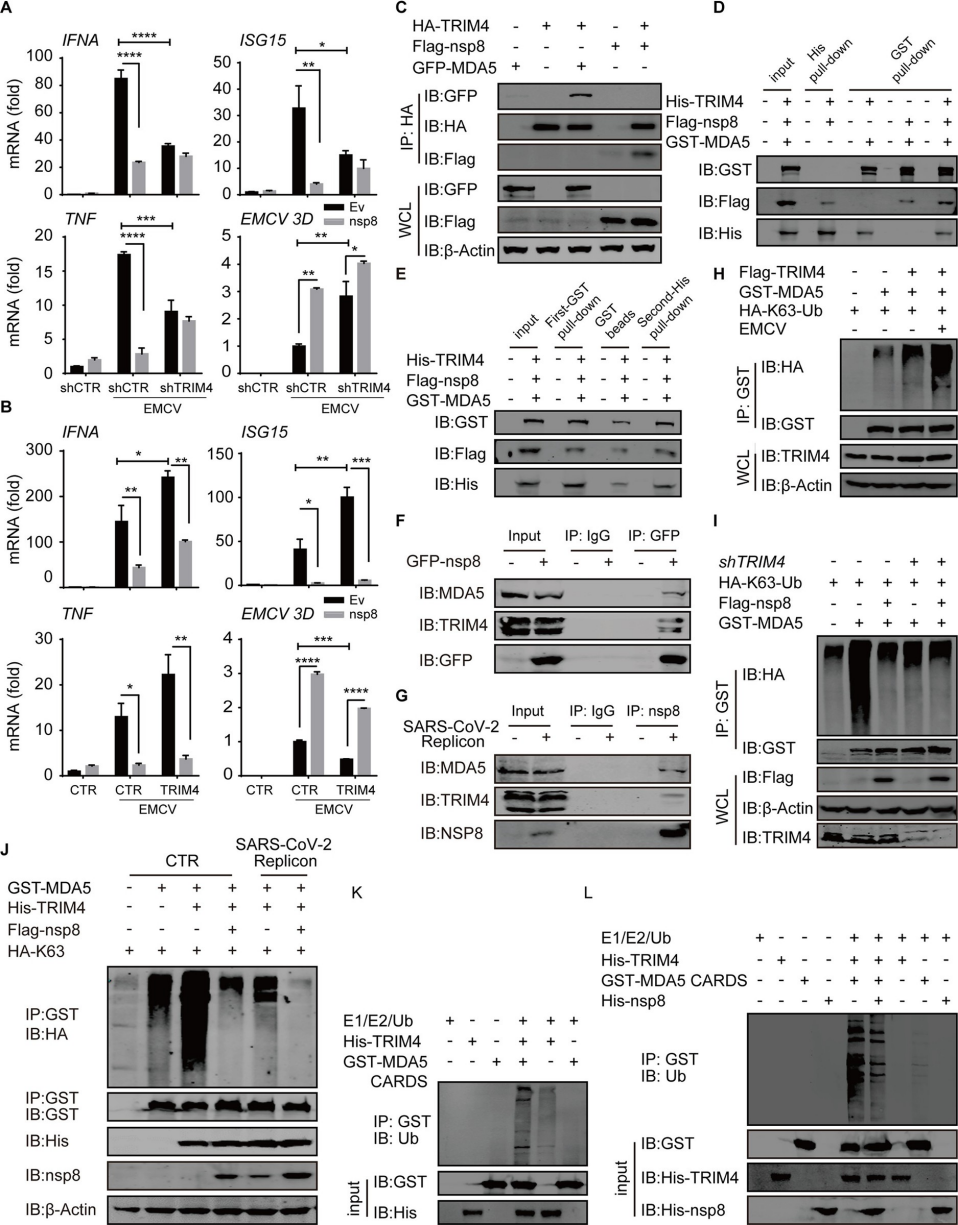
TRIM4 is required for MDA5 K63-linked polyubiquitination and Nsp8 inhibition in antiviral responses. A-B. HEK293T cells were co-transfected with an empty vector or nsp8-expressing plasmid, plus scramble shRNA or shTRIM4 (A), or empty vector or TRIM4-expressing plasmid (B) for 24 h, and then left uninfected or infected with EMCV (MOI = 0.25) for 18 h. The total RNA was extracted, reverse-transcribed and detected with real-time PCR analysis for *IFNA*, *ISG15*, *TNF* and *EMCV 3D* gene expression. The results are shown as the mean ± SD (n = 3), *, p < 0.05; **, p < 0.01; ***, p < 0.001; ****, p < 0.0001, by Sidak’s multiple comparisons test. C. Flag-nsp8, GFP-MDA5 and/or HA-TRIM4-expressing plasmids were co-transfected into HEK293T cells as indicated for 36 h. Then cell lysates were subjected to coimmunoprecipitation with anti-HA beads, followed by immunoblotting with the indicated antibodies. D-E. GST or His-tagged fusion proteins were immobilized on GST-beads or Ni-NTA resins as indicated, and then incubated with Flag-nsp8 protein purified from lysates of HEK293T cells for 2 hours(D). For sequential double pull-down assays, the glutathione agarose beads were eluted with GSH solution(E). After five washing, the beads were analyzed by immunoblotting analysis. F-G. HEK293T cells were transiently transfected with GFP-nsp8 expressing plasmids (F) or Spike-deleted SARS-CoV-2 replicon BAC DNA (G) for 36h. The cell lysates were subjected to coimmunoprecipitation using anti-GFP beads (F) or anti-nsp8 antibody plus protein A/G beads (G), and analyzed by western blotting as indicated to detect endogenous MDA5 and TRIM4. H. GST-MDA5 and Flag-TRIM4-expressing plasmids were co-transfected into HEK293T cells with HA-K63-Ub expressing plasmid, followed with uninfected or infected with EMCV (MOI = 0.25) for 18 h. The cells were collected, lysed and subjected to ubiquitination assay with immunoprecipitation with anti-GST beads and western blotting with antibodies as indicated. I. Flag-nsp8 and GST-MDA5 expressing plasmids were co-transfected into HEK293T cells with scramble shRNA or shTRIM4 as indicated. The cell extracts were subjected to in vivo ubiquitination assay as indicated. J.GST-MDA5, His-TRIM4 and/or Flag-nsp8 plasmids were co-transfected with HA-K63-Ub expressing plasmid into uninfected HEK293T cells or cells harboring SARS-CoV-2 replicon for 36h. The K63-polyubiquitination of MDA5 was analyzed as described above. K-L. Purified GST-tagged MDA5 CARDs protein was bound to GST-beads and then incubated for 1 h at 37°C with TRIM4 protein (K) and/or nsp8 protein (L) in in vitro ubiquitination reaction. The beads were washed three times with PBS containing 0.1% SDS and 0.2% Triton X-100 and then immunoblotted with antibodies to Ub.

To further investigate the suppression of MDA5 K63-linked polyubiquitination, the roles of several E3 ligases in nsp8-mediated inhibition of MDA5 antiviral responses were investigated (S5A and S5B Fig). TRIM65 and TRIM13 play critical roles in MDA5-mediated antiviral immune responses [35,36], however, depletion of TRIM13 enhanced MDA5-induced ISRE activity but did not attenuate the inhibitory effect of nsp8 on MDA5-induced ISRE activation. Similarly, TRIM25 or TRIM65 depletion decreased MDA5-induced ISRE activity but did not attenuate the inhibitory effect of nsp8. These results suggest that none is the direct target of nsp8-mediated MDA5 inhibition. After we searched the public sources for viral protein-protein interactions [37], four nsp8-binding E3 ligases were depleted and the effects evaluated, and we found that silencing of TRIM4 significantly decreased MDA5-induced ISRE activity and almost completely abolished the inhibitory effect of nsp8. Indeed, MDA5-mediated immune and inflammatory gene expression including *IFNA*, *ISG15* and *TNF* was dramatically inhibited by nsp8 overexpression under EMCV-infection, and TRIM4 silencing decreased the expression levels of these genes and almost completely abolished the inhibitory effect in the presence of nsp8 expression (Fig 5A). In contrast, the expression levels of these genes were increased by TRIM4 overexpression, and nsp8 exhibited obvious inhibitory effects (Fig 5B). Consequently, EMCV infection was enhanced by TRIM4 depletion while it was inhibited by TRIM4 overexpression, and nsp8 greatly promoted viral replication under both conditions (Fig 5A and 5B). These results suggest that nsp8 suppresses MDA5-mediated antiviral immune responses in TRIM4-dependent manner.

Next, we investigated whether nsp8 inhibits TRIM4-induced K63-linked polyubiquitination of MDA5. Coimmunoprecipitation analyses showed that TRIM4 interacted with both MDA5 and nsp8 (Fig 5C), and their interaction did not affect each other and MDA5 depletion also did not affect nsp8-TRIM4 interaction (S5C–S5E Fig). Furthermore, their purified proteins were mixed with each other, and a pull-down assay confirmed that they directly interacted with each other in vitro (Fig 5D). Moreover, a sequential double pull-down assay showed that nsp8, TRIM4 and MDA5 formed trimeric complexes together (Fig 5E). Importantly, both ectopic and SARS-CoV-2-expressing nsp8 obviously interacted with endogenous TRIM4 and MDA5 (Fig 5F and 5G). These results suggest that nsp8 interacts with TRIM4 and MDA5 in cells infected with SARS-CoV-2. We further investigated the polyubiquitination of MDA5 by TRIM4 in the absence or presence of nsp8. Although the association between TRIM4 and MDA5 was not affected by EMCV infection (S5F Fig), TRIM4 induced MDA5 K63-linked polyubiquitination and EMCV infection augmented it (Fig 5H). Following the increasing expression of nsp8 in HEK293T cells, auto-polyubiquitination of TRIM4 was enhanced (S5G Fig), suggesting that nsp8 may affect the catalytic activity of TRIM4, probably delaying ubiquitination and then leading to the accumulation of polyubiquitin chains linked with TRIM4. Importantly, either nsp8 overexpression or TRIM4 depletion decreased MDA5 K63-linked polyubiquitination, and the inhibitory effect of nsp8 on MDA5 polyubiquitination was almost completely abolished under TRIM4 silencing (Fig 5I), indicating that TRIM4 was required for MDA5 K63-linked polyubiquitination and nsp8-mediated inhibition. As a result, TRIM4-mediated MDA5 K63-linked polyubiquitination was suppressed by SARS-CoV-2 infection that expressed endogenous nsp8, and ectopic nsp8 overexpression augmented the inhibition (Fig 5J). To further confirm that MDA5 was directly ubiquitinated by TRIM4, we performed an in vitro ubiquitination assay using purified MDA5 CARDs in the absence or presence of TRIM4. In vitro polyubiquitination of MDA5 CARDs was observed in the presence of TRIM4 but not in the absence of TRIM4 in a mixture of E1 and E2 enzymes and ubiquitin (Fig 5K). The TRIM4-mediated in vitro polyubiquitination of MDA5 CARDs was greatly decreased in the presence of nsp8 (Fig 5L). These results suggest that TRIM4 interacts with MDA5 and mediates its K63-linked polyubiquitination while nsp8 inhibits TRIM4-mediated MDA5 K63-linked polyubiquitination during viral infection.

**Fig 6:**
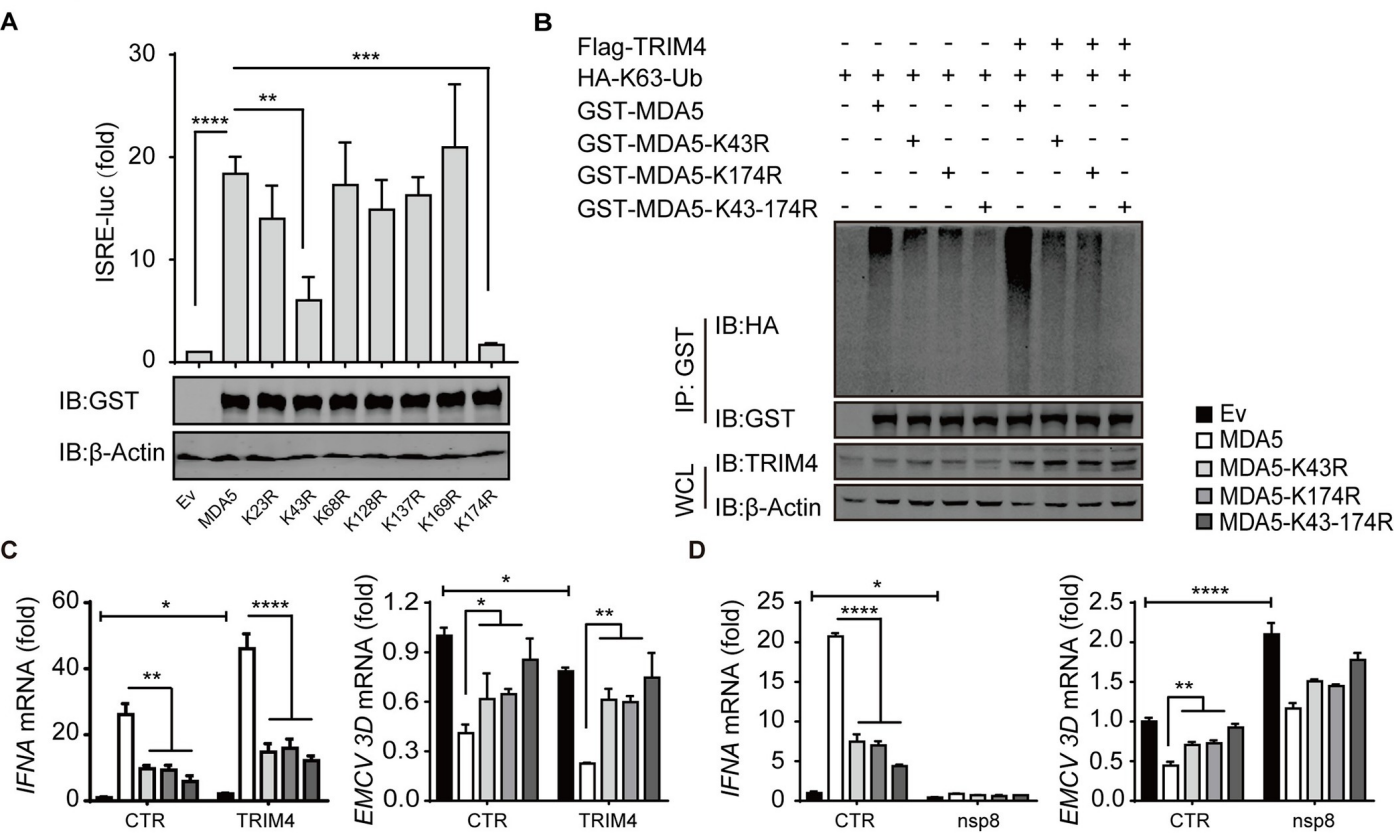
TRIM4-mediates MDA5 K63-linked polyubiquitination at K43 and K174 residues. A. The plasmids of wild type GST-MDA5 or single-point mutation of lysine residue in MDA5 CARDs were co-transfected into HEK293T cells with ISRE-firefly luciferase reporter, and renilla luciferase as internal control. Then the cells were collected and the luciferase-based ISRE activity were measured, the expression levels of wild type or mutated MDA5 was detected by western blotting. The results are shown as the mean ± SD (n = 3), **, p < 0.01; ***, p < 0.001; ****, p < 0.0001, by Sidak’s multiple comparisons test. B. Wild type or K43R or K174R or K43R-174R mutated GST-MDA5 expressing plasmids were co-transfected into HEK293T cells with vector or Flag-TRIM4 expressing plasmid and HA-K63-Ub construct as indicated. The cell extracts were subjected to in vivo ubiquitination assay as indicated. C-D. Wild type or K43R or K174R or K43R-174R mutated MDA5 expressing plasmids were transfected into HEK293T in presence of vector or TRIM4 overexpression (C), vector or nsp8 overexpression (D). Twenty-four hours later, the cells were infected with EMCV (MOI = 0.25) for additional 18 h and then total RNA were extracted, reverse-transcribed and detected with real-time PCR analysis for antiviral gene expression and viral replication. The results are shown as the mean ± SD (n = 3), *, p < 0.05; **, p < 0.01; ***, p < 0.001; ****, p < 0.0001, by Sidak’s multiple comparisons test and multiple t-tests.

To further characterize the sites of K63-linked polyubiquitination in MDA5, the lysine residues in MDA5 CARDs were individually mutated to arginine, and two single-point mutants (K43R and K174R) significantly decreased MDA5-induced ISRE activity (Fig 6A). Both single-point K43R and K174R mutations greatly decreased and double-point K43R-K174R mutation almost completely abolished MDA5 K63-linked polyubiquitination under endogenous expression conditions or in the presence of TRIM4 overexpression (Fig 6B), indicating that TRIM4 induced MDA5 K63-linked polyubiquitination at both K43 and K174 residues. Furthermore, either mutation in MDA5 decreased the induction of *IFNA* gene expression in both the absence and presence of TRIM4 overexpression and consequently attenuated the inhibitory effects on viral infection and replication, and double-point mutation declined more (Fig 6C); in addition, nsp8 inhibited *IFNA* gene expression and increased viral gene expression regardless of whether wild-type or mutated MDA5 was overexpressed (Fig 6D). These results indicate that nsp8 suppresses TRIM4-mediated K63-linked polyubiquitination of MDA5 and then inhibits MDA5-mediated antiviral immune responses.

**Fig 7:**
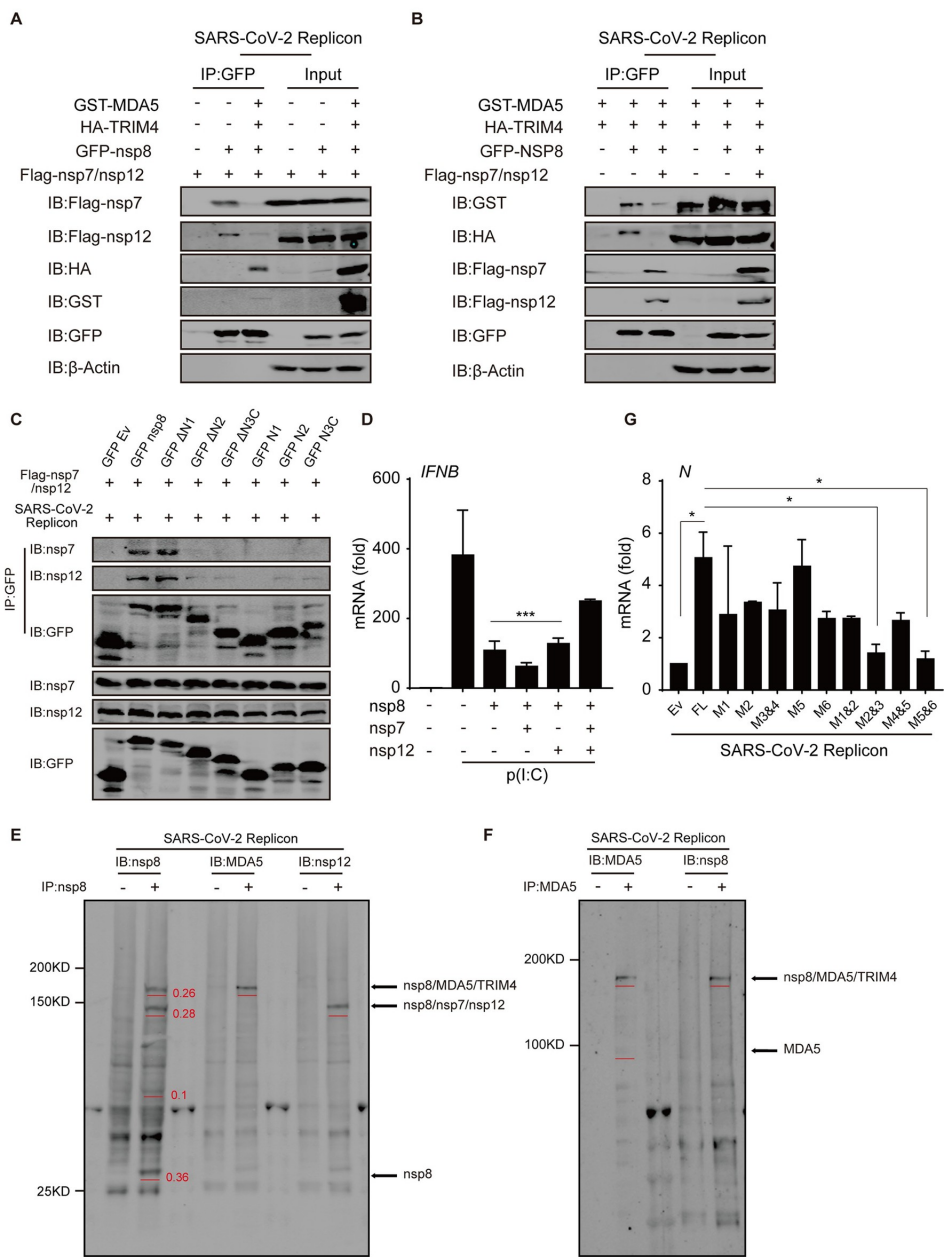
Nsp7/Nsp8/Nsp12 competitively suppresses Nsp8 inhibitory function in antiviral responses. A-B. HEK293T cells harboring SARS-CoV-2 replicon were co-transfected with GFP-nsp8, GST-MDA5 or HA-TRIM4 plasmids in presence of Flag-nsp7/nsp12 overexpression (A), or GFP-nsp8 or Flag-nsp7/nsp12 plasmids in presence of GST-MDA5 and HA-TRIM4 overexpression (B) as indicated. 36 h post transfection, the cells were collected and whole cell extracts were subjected to co-immunoprecipitation with anti-GFP beads, followed with western blotting analysis as indicated. C. HEK293T cells harboring SARS-CoV-2 replicon were transfected with GFP-nsp8 and its truncated mutants along with Flag-nsp7/nsp12 expressing plasmids. After 36 h, the cells were collected and cell lysates were subjected to coimmunoprecipitation using anti-GFP beads and followed by western blotting analysis with the indicated antibodies. D. HEK293T cells were co-transfected with empty vector or nsp8-expressing plasmids with nsp7 and/or nsp12 expressing plasmids alone or together. Twenty-four hours post transfection, cells were stimulated with poly(I:C) (5 μg/ml) for 24 h and total RNA was extracted and subjected to RT-PCR analysis for *IFNB* expression. The results are shown as the mean ± SD (n = 3), ***, p < 0.001, by Sidak’s multiple comparisons test. E-F. HEK293T cells harboring SARS-CoV-2 replicon were collected, the cell extracts were cross-linked with 2 mM DSS for 30 min and quenched with 50 mM Tris-HCl (pH = 8.0) for 15min and then subjected to immunoprecipitation with IgG control or mouse anti-nsp8 antibody (E), or IgG control or rabbit anti-MDA5 antibody (F), and immunoprecipitated complexes were analyzed by western blotting with rabbit anti-nsp8, anti-MDA5 or anti-nsp12 antibody (E), or mouse anti-MDA5 or anti-nsp8 antibody (F). The specific bands were labeled and the relative proportions were calculated based on the grayscale intensity of bands and shown. G. Empty vector or nsp8 point-mutants were transfected into HEK293T cells harboring SARS-CoV-2 replicon. Thirty-six hours post transfection, total RNA was extracted and subjected to RT-PCR analysis for viral gene *N* expression. The results are shown as the mean ± SD (n = 3), *, p < 0.05, by Sidak’s multiple comparisons test.

As nsp8 is an important component of the viral replication complex (nsp7/nsp8/nsp12) [34,38], nsp8 may exist in two different complexes, namely, the nsp7/nsp8/nsp12 complex and the nsp8/TRIM4/MDA5 complex, in SARS-CoV-2-infected cells. We further investigated whether these complexes influence each other. In Spike-deficient SARS-CoV-2-infected cells, ectopic TRIM4 and MDA5 expression suppressed the association of nsp8 with nsp7/nsp12 (Fig 7A). In contrast, nsp7/nsp12 overexpression decreased the interaction of nsp8 with TRIM4/MDA5 (Fig 7B). These results suggest that nsp8 in the immunomodulatory complex and that in the viral replication complex competitively suppress the function of the other. Computer-based structural analysis showed that the C-terminal domain of nsp8 was critical for MDA5 inhibition (Fig 3C) and the nsp7/nsp12 interaction [33,34], and deletion of this domain abrogated the interaction of nsp8 with nsp7/nsp12 (Fig 7C); thus, MDA5 and nsp7/nsp12 may repulse each other because of space constrains. As a result, nsp7/nsp8/nsp12 overexpression almost abolished the inhibitory effect of nsp8 on IFN expression after poly(I:C) stimulation, even though nsp7 alone augmented and nsp12 alone negligibly affected IFN expression (Fig 7D). These results suggest that nsp8 inhibits antiviral immune responses through the nsp8/TRIM4/MDA5 complex but not the viral replication complex.

To further detect the relative proportions of different nsp8 complexes in SARS-CoV-2 infected cells, the endogenous nsp8 protein complexes were cross-linked with DSS and immunopreciptiated with anti-nsp8 antibody. Immunoblotting analysis show that four main kinds of nsp8 exist in SARS-CoV-2 replicon harboring cells: free nsp8, nsp7/nsp8/nsp12, nsp8/TRIM4/MDA5 and an unknown nsp8 complex, the relative proportions of nsp8 is: free nsp8 > nsp7/nsp8/nsp12 > nsp8/TRIM4/MDA5 > the unknown nsp8 complex, based on the intensity of nsp8 band in different complexes (Fig 7E). The results show that both endogenous nsp7/nsp8/nsp12 and nsp8/TRIM4/MDA5 complexes naturally exist in SARS-CoV-2 infected cells and appropriately one-third free nsp8 exists rather than that nsp8 mainly exists in nsp7/nsp8/nsp12 complex. Similarly, we determined the proportion of MDA5 in SARS-CoV-2 replicon infected cells, and found that MDA5 mainly exists in nsp8/TRIM4/MDA5 complex and very weak free MDA5 was detected (Fig 7F). Given that a high proportion of free nsp8 exists in SARS-CoV-2 infected cells, the results reveal that endogenous MDA5 mainly exists in nsp8/TRIM4/MDA5 complex and is suppressed by nsp8 during SARS-CoV-2 infection. These results suggest that endogenous nsp8 indeed interacts with MDA5 and TRIM4 to suppress MDA5 innate immune responses in SARS-CoV-2 infected cells.

### Nsp8 suppresses antiviral responses and promotes SARS-CoV-2 infection in MDA5-dependent manner

**Fig 8:**
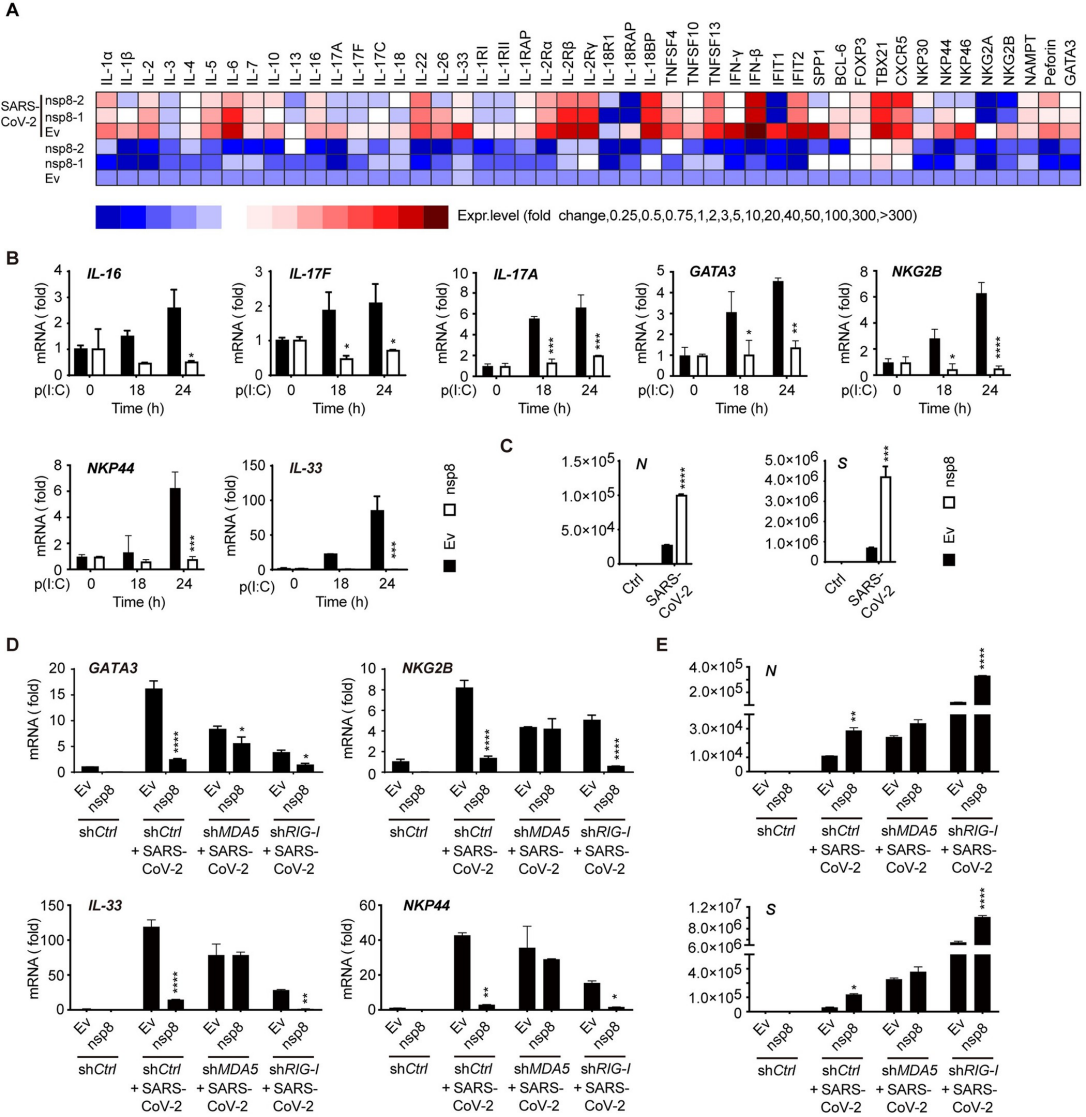
Nsp8 decreases antiviral immune and inflammatory responses and promotes SARS-CoV-2 infection. A. A549 cells were transfected with an empty vector or Flag-nsp8 expressing plasmid along with Flag-ACE2 expressing plasmid. Twenty-four hours post transfection, cells were left untreated or infected with SARS-CoV-2 (MOI = 3) for 18 h, and then cells were collected, and total RNA was extracted and subjected to RT-PCR analysis for the expression of the indicated genes. B. A549 cells were transfected with an empty vector or Flag-nsp8 expressing plasmid. 24 h post transfection, cells were treated with poly(I:C) (5 μg/ml) for the indicated time points, and then total RNA was extracted and subjected to RT-PCR analysis of the expression of selected genes *IL-16*, *IL-17F*, *IL-17A*, *GATA3*, *NKG2B*, *NKP44* and *IL-33*. The results are shown as the mean ± SD (n = 3), *, p < 0.05; **, p < 0.01; ***, p < 0.001; ****, p < 0.0001, by Sidak’s multiple comparisons test. C. A549 cells were transfected with a control vector (Ev) or Flag-nsp8 expressing plasmid along with Flag-ACE2 expressing plasmid. Twenty-four hours post transfection, cells were infected with SARS-CoV-2 (MOI = 3) for 18 h and then subjected to RT-PCR analysis for viral genes *N* and *S* expression. The results are shown as the mean ± SD (n = 3), ***, p < 0.001; ****, p < 0.0001, by Sidak’s multiple comparisons test. D. Control (shCtrl), MDA5 knockdown (shMDA5) or RIG-I knockdown (shRIG-I) HEK293T cells were transfected with an empty vector or Flag-nsp8 along with Flag-ACE2 expressing plasmid. Twenty-four hours post transfection, cells were infected with SARS-CoV-2 (MOI = 3) for 18 h and then total RNA was extracted and subjected to RT-PCR analysis for *GATA3*, *NKG2B*, *NKP44* and *IL-33* expression. The results are shown as the mean ± SD (n = 3), *, p < 0.05; **, p < 0.01; ***, p < 0.001; ****, p < 0.0001, by Sidak’s multiple comparisons test and multiple t-tests. E. Control (shCtrl), MDA5 knockdown (shMDA5) or RIG-I knockdown (shRIG-I) HEK293T cells were transfected with an empty vector or Flag-nsp8 along with Flag-ACE2 expressing plasmid. Twenty-four hours post transfection, cells were treated with SARS-CoV-2 (MOI = 3) for 18 h and then total RNA was extracted and subjected to RT-PCR analysis for *N*, *S*. The results are shown as the mean ± SD (n = 3), *, p < 0.05; **, p < 0.01; ***, p < 0.001; ****, p < 0.0001, by Sidak’s multiple comparisons test.

Next, we investigated whether nsp8 affects the downstream expression of immune and inflammatory genes. After the cells were left uninfected or infected with SARS-CoV-2, nsp8-expressing A549 cells were collected, and total RNA was subjected to RT-PCR array analysis for immune and inflammatory gene expression. Infection of SARS-CoV-2 greatly induced the expression of many cytokines and chemokines and receptors, while the expression of the majority of cytokines, including *IL-1β*, *IL-2*, *IL-5*, *IL-6*, *IL-26*, *IL-33*, *IFNB*, *IFIT1* and *IFIT2*, was downregulated by nsp8 expression. The transcription of some pleiotropic chemoattractant cytokines, such as *IL-16*, *IL-17A*, *IL-17F*, and *IL-17C*, was also downregulated. Furthermore, a decreasing transcription tendency was also observed for the inflammatory receptors *IL-1RI*, *IL-1RII*, *IL-2Rα*, and *IL-18RAP*; NK cell-associated activation receptors, such as *NKp44*, *NKp46*, and *NKG2B*; and the trans-acting T-cell-specific transcription factor *GATA3* (Fig 8A), indicating that the activation of T cells and NK cells was attenuated by nsp8 through the suppression of the gene expression of these key factors. Although these decreased cytokines and receptors may not be directly activated by IRF3 or NF-κB pathways, they could be regulated by downstream cytokines or other factors derived from these two pathways. In contrast, the cytokine IL-2 and IFN-gamma suppression gene *FOXP3* was significantly increased with nsp8 overexpression. Similarly, the levels of these cytokines and inflammatory factors were downregulated by nsp8 overexpression during SARS-CoV-2 infection in A549 cells (Fig 8A). Collectively, these results suggest that nsp8 could strongly impair the expression of genes involved in antiviral immune and inflammatory responses. To further confirm that the downregulation of these immune and inflammatory cytokines and genes is mediated by nsp8 under physiological conditions, A549 cells were transfected with a nsp8-expressing plasmid or empty vector and then stimulated IC poly(I:C) mimicking viral RNA for the indicated times. The inhibition of the expression of key cytokines and related genes was verified, and nsp8 negatively regulated the expression of these immune and inflammatory genes (Figs 8B and S6). Collectively, these results suggest that nsp8 could strongly impair the expression of genes involved in antiviral immune and inflammatory responses.

The infectious activities of SARS-CoV-2 were then assessed. nsp8 overexpression in A549 cells significantly upregulated viral *N* and *S* expression after challenge with SARS-CoV-2 (Fig 8C), indicating that antiviral activities were greatly weakened by nsp8 expression and that nsp8 consequently promotes SARS-CoV-2 infection and replication. Although the point-mutations of nsp8 at paired sites that interact with MDA5 did not greatly attenuate the inhibitory effect on antiviral immune responses (S3E Fig), overexpression of these mutated nsp8 differently regulated viral RNA level, two-point mutation A76G/E79A or Q90A/I122A significantly decreased the function in viral replication while other mutations slightly affected it (Fig 7G), indicating that nsp8 suppresses antiviral innate immune responses independent on its function in viral replication.

Consistent with previous results showing that the suppression of antiviral responses by nsp8 was dependent on MDA5 (Fig 2H–2M), MDA5 knockdown was performed and blocked the inhibitory effect of nsp8 on the expression of *GATA3*, *NKG2B*, *IL-33* and *NKP44* during SARS-CoV-2 infection, while RIG-I knockdown cells exhibited intact nsp8-mediated inhibition of the expression of these genes (Fig 8D). Importantly, the infectious activities of SARS-CoV-2 were greatly enhanced by either MDA5 depletion or RIG-I depletion, and viral *N* and *S* expression was augmented by nsp8 in RIG-I-depleted cells, while neither gene was upregulated in the presence of nsp8 expression under MDA5 depletion (Fig 8E). In conclusion, these results show that nsp8 overexpression favors SARS-CoV-2 infection by inhibiting MDA5-mediated antiviral responses.

## Discussion

To explore the roles of SARS-CoV-2 proteins, especially the nonstructural protein family (NSP), in viral immune evasion, we screened a panel of NSPs using ISRE-luciferase reporter and identified nsp8 as an immune suppressor of the type I IFN signaling pathway in the present study. We have shown that nsp8 overexpression impaired type I IFN production and the gene expression of immune and inflammatory factors, including *TNF*, *IFNB*, *IFIT1*, *IFIT2*, *IL-6* and *CCL20*. As a consequence, we observed that SARS-CoV-2 infection and replication was significantly increased in cells with nsp8 overexpression. The subsequent detection of phosphorylation of kinases TBK1 and IKKα/β and substrates IRF3 and p65 provided evidence that both IRF3 and NF-κB activity were inhibited by nsp8. Consequently, a series of antiviral immune and inflammatory cytokines and related genes were further strongly downregulated by nsp8 expression. Furthermore, SARS-CoV-2 infection in the presence of MDA5- or RIG-I silencing showed that nsp8 suppresses antiviral immune and inflammatory responses in an MDA5-dependent manner.

Several studies have shown that SARS-CoV-2 infection are sensed by both RIG-I-like receptors and MDA5 acts as a main sensor of SARS-CoV-2 [8,31,32,39]. Herein, we speculated that nsp8 may acts as a direct suppressor on MDA5, the upstream viral dsRNA sensors. Our results showed that nsp8 directly interacted with MDA5 but not RIG-I on its CARDs domains, and MDA5-mediated antiviral immune and inflammatory responses were strongly inhibited at the same time. Nsp8 greatly inhibits *TNF* and *IFNB* expression under EMCV infection and EMCV RNA stimulation as specific stimuli of MDA5, and nsp8-mediated inhibition of *TNF* and *IFNB* was almost completely abolished by MDA5 depletion but not by RIG-I depletion in the presence of EMCV RNA stimulation or EMCV infection (Fig 2I and 2M), indicating that nsp8 suppresses antiviral immune and inflammatory responses through the MDA5 pathway. In addition, nsp8 does not function as the main IFN regulator and barely exhibits an impact on interferon responses under RIG-I specific stimulation (Fig 2H and 2L), underlining the specific inhibitory effect of nsp8 on the MDA5 signalosome.

The MDA5-MAVS axis can activate both the IRF3 and NF-κB pathways through the phosphorylation of IKKα/β and TBK1, and our results revealed that nsp8 inhibits the phosphorylation of IKKα/β, TBK1, IRF3 and NF-κB p65 (Fig 2C–2E). As a result, nsp8 suppresses both *IFNB* and *TNF* expression during both EMCV and SARS-CoV-2 infection in MDA5-dependent manner (Fig 2I and 2J), to facilitate SARS-CoV-2 infection and replication. On the other hand, prolonged NF-κB activation is also regulated by several viral proteins and may be essential for SARS-CoV-2 infection and replication [40–44]. It is important to detect MDA5-mediated *TNF* expression in the early stage of infection with high viral titer (MOI = 3 for 18 h) to ensure that MDA5 rapidly senses viral dsRNA and then activates the NF-κB pathway. In contrast, at the late stage of infection with low viral titer (MOI = 0.1 for 48 h), viral proteins and their downstream signaling can trigger NF-κB activation and *TNF* expression in MDA5-independent manner, which depends on viral gene expression and replication [32]. Thus, different dynamics of NF-κB activation and TNF responses induced by SARS-CoV-2 infection depend on the cell type, viral titer and time course.

Polyubiquitinated modification of MDA5 is crucial for its antiviral responses, K48-linked polyubiquitination mediates MDA5 proteasomal degradation [45], and K63-linked polyubiquitination mediates MDA5-induced type I IFN expression [13]. We speculated that nsp8 may inhibit type I IFN signaling through the polyubiquitinated modification of MDA5. To test our hypothesis, we determined the status of WT-, K48- and K63- linked polyubiquitination of MDA5 in the absence or presence of nsp8 and confirmed that WT- and K63- linked polyubiquitination were impaired by nsp8, while K48-linked polyubiquitination was barely changed (Fig 4A–4C). We therefore conclude that under certain circumstances, nsp8 jeopardizes antiviral responses by impairing MDA5 K63-linked polyubiquitination.

Studies have shown that K63-linked polyubiquitination of MDA5 CARDs probably at K174 residue is essential for the activation of type I IFN expression [13]. However, the E3 ubiquitin ligase responsible for MDA5 K63-polyubiquitination has not been characterized. Although TRIM13 and TRIM65 interact with MDA5 and regulate its K63-polyubiquitination [35,36], their depletion did not abolish nsp8-mediated inhibition of MDA5 signalosome (S5A and S5B Fig). In the present study, we reveal that TRIM4 positively regulates MDA5–mediated antiviral immune responses by inducing MDA5 K63-linked polyubiquitination at K43 and K174, depletion of TRIM4 or mutation of MDA5 at both sites greatly suppresses MDA5 K63-polyubiquitination and antiviral innate immune responses. Therefore, there are two different mechanisms of K63-linked polyubiquitination in MDA5 activation: unanchored binding of long K63-linked polyubiquitin chains bridges MDA5 and MAVS for complex assembly and signal transduction [11], and TRIM4 induces the covalent linkage of K63-linked polyubiquitin chains to K43 and K174 in the MDA5 CARDs for activation of antiviral immune responses.

Our results revealed that nsp8 interacts with TRIM4 and suppresses TRIM4-mediated MDA5 K63-linked polyubiquitination for innate immune inhibition (Figs 5 and 6), and TRIM4 silencing almost completely abolishes nsp8-mediated inhibition of MDA5 K63-linked polyubiquitination and antiviral responses during infection with dsRNA viruses. TRIM4 also interacts with RIG-I and induces its K63-linked polyubiquitination to activate antiviral immune responses [46]; we observed that nsp8 weakly interacts with RIG-I and slightly inhibited RIG-I-mediated antiviral responses (Figs 2A and 3A); nsp8 probably also downregulates RIG-I polyubiquitination by binding competitively to TRIM4 and/or inhibiting its catalytic activity. These results provide evidence that TRIM4 functions as the direct target of nsp8 in evasion of antiviral immune responses during SARS-CoV-2 infection by impairing K63-linked polyubiquitination of two RNA sensors MDA5 and RIG-I, preferentially with a higher affinity for and main inhibitory activity towards MDA5 signalosome.

**Fig 9:**
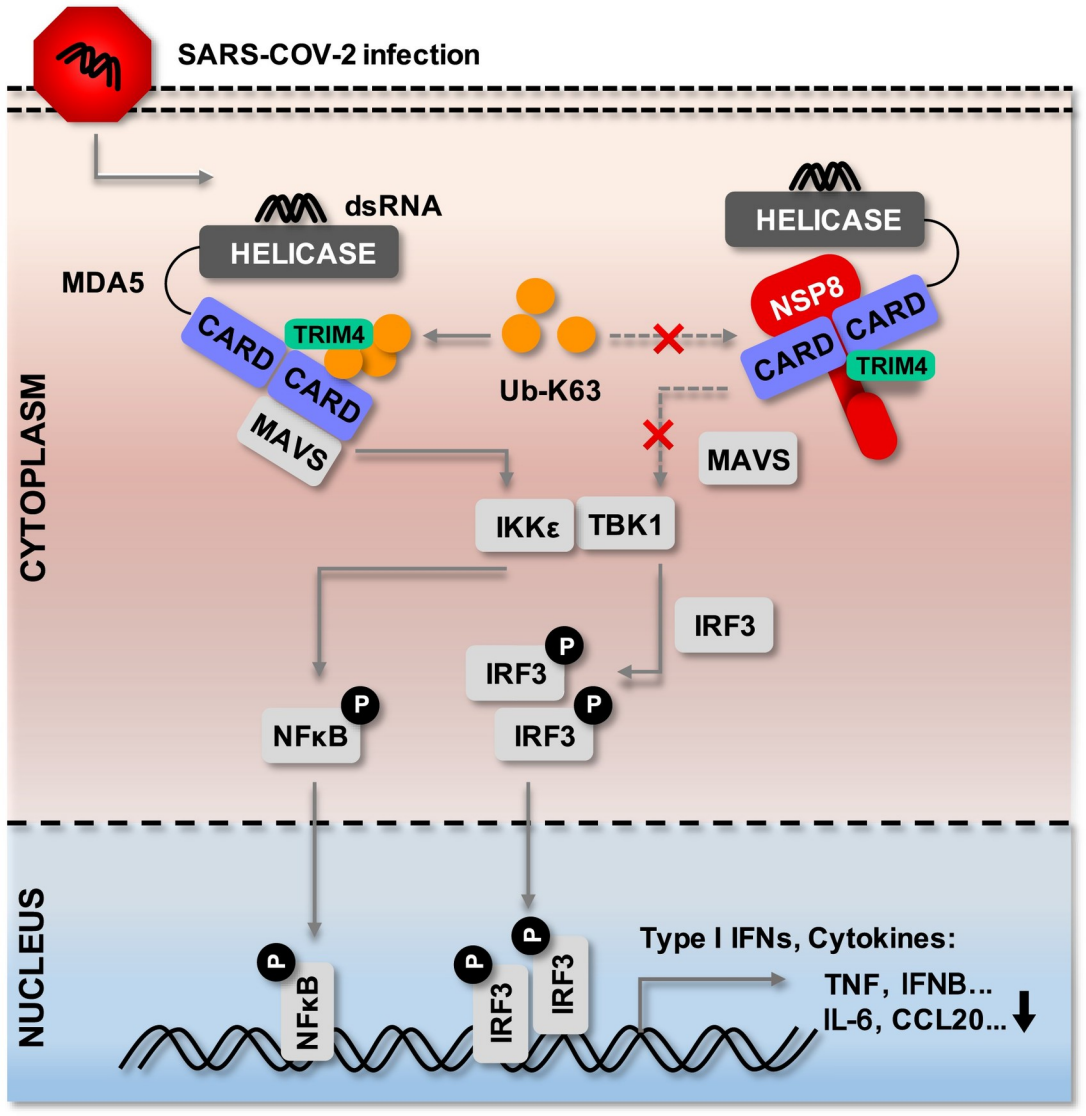
Working model of SARS-CoV-2 Nsp8 negative regulation on MDA5-mediated antiviral responses. During SARS-CoV-2 infection, nsp8 interacts with TRIM4 and MDA5 CARDs domain and then impairs TRIM4-mediated K63-linked polyubiquitination, thus attenuates the recruitment of MAVS and subsequently inhibits the phosphorylation of TBK1, NF-κB and IRF3, and then downregulates the production of type I IFNs, cytokines and inflammatory factors, therefore facilitates SARS-CoV-2 infection and replication.

Based on our existing experimental data, we propose a simple working model to illustrate how nsp8 negatively regulates innate immune responses by binding to TRIM4 and MDA5 and then inhibiting TRIM4-mediated MDA5 K63-linked polyubiquitination (Fig 9). Upon SARS-CoV-2 infection, cytosolic viral dsRNA is recognized by MDA5 and triggers the expression of type I IFN and cytokines; moreover, nsp8 is highly expressed and localized in the cytoplasm of host cells and then interacts with MDA5 CARDs and TRIM4 to form a trimeric complex to shield MDA5 from ubiquitin chains and inhibit TRIM4-mediated K63-linked polyubiquitination, consequently negatively regulates type I IFN signaling and antiviral immune responses, thus favors viral infection and replication.

Interestingly, a recent study showed that the MDA5 gene of pangolin (the carrier and intermediate host of coronaviruses) is mutated and dysfunctional [47], while RIG-I and TLR 3, 7 and 8 are conserved among three different species. Considering the coexisting relationship between pangolins and coronaviruses and the tolerance of pangolins for SARS-CoV-2 infection, this finding implied that coronaviruses may be inclined to silence MDA5-mediated innate immunity because they could generate more drastic reactivity and injury upon viral RNA recognition. Based on this observation and the findings of our present study, the suppressive function of nsp8 in antiviral responses and its impairment of MDA5 activation have been identified, representing a direct immune evasion mechanism of the recognition of viral dsRNA.

Multiple posttranslational modifications, including phosphorylation, acetylation, methylation and polyubiquitination, are employed to regulate the antiviral signalosome [48]. Among these modifications, polyubiquitination is commonly used for the degradation or activation of MDA5 and RIG-I. The E3 ubiquitin ligase TRIM40 targets MDA5 and RIG-I to promote their K27- and K48- linked ubiquitination, thus leading to their proteasomal degradation for immune silencing. Upon RNA virus infection, TRIM40 is downregulated to allow the activation of a sufficient antiviral immune response [45]. Likewise, some viral proteins, such as Epstein-Barr virus protein BPLF1, which has ubiquitin- and NEDD8-specific deconjugase activity, interact with scaffold proteins 14-3-3 and TRIM25 to form a tri-molecular complex, consequently promoting the dimerization and ubiquitination of TRIM25. Consequently, K63-linked polyubiquitination of RIG-I is downregulated, leading to the attenuation of RIG-I-mediated type I IFN antiviral responses [49]. In addition, the NS3 protein of ZIKA virus interacts with scaffold proteins 14-3-3ϵ and η separately through its 14-3-3 binding motif, hence blocking the translocation of RIG-I and MDA5 from the cytosol to mitochondria, impairing signalosome formation with MAVS, and antagonizing innate immunity [50]. Our studies revealed that nsp8 of SARS-CoV-2 acts as a binding partner of MDA5 to shield its K63-linked polyubiquitination and impairs the formation or activation of the MDA5 signalosome. This finding might represent a novel immune evasion strategy of SARS-CoV-2 infection by directly impairing the activation of the main viral RNA sensor MDA5.

In summary, our study provides insights into the potential mechanisms by which SARS-CoV-2 nsp8 inhibits type I IFN signaling and antiviral responses. We provide compelling evidence that nsp8 plays a critical negative role in TRIM4- and MDA5- mediated antiviral responses and demonstrate specific modulation of the viral dsRNA-triggered signalosome and signal cascade by nsp8. Importantly, considering that MDA5 plays a key pathological role in antiviral immunity toward severe coronaviruses, antagonists of nsp8 could serve as promising therapeutic agents for COVID-19 therapies.

## Materials and methods

### Cell cultures, antibodies and reagents

HEK293T, Hela and Vero cells were cultured in DMEM (KeyGEN BioTECH) medium supplemented with 10% FBS (Certified Fetal Bovine Serum; VivaCell, Shanghai, China) and 1% antibiotics (penicillin and streptomycin). A549 cells (human adenocarcinoma lung tissue-derived epithelial cells) were cultured in RPMI 1640 (Gibco) medium containing 10% FBS and 1% antibiotics.

The following antibodies and reagents were used in this study: goat anti-mouse IRDye680RD (C90710-09) and goat anti-rabbit IRDye800CW (C80925-05), purchased from Li-COR; anti-HA (M132-3) and anti-Flag (M185-3L), purchased from MBL; anti-nsp8 (GTX632696, GTX636997) and anti-ISG15 (GTX121474), purchased from GeneTex; anti-GFP (AE011), anti-nsp7 (A20201), anti-nsp8 (A20202), anti-nsp12 (A20233), anti-RIG-I/DDX58 (A0550), anti-MDA5/IFIH1 (A13645) and anti-IRF3 (A2172), purchased from ABclonal; anti-Tubulin (HC101-01), purchased from TransGen; anti-Ubiquitin(#3936), anti-pIKKα/β(#2697), anti-pTBK1 (#5483), anti-TBK1 (#3504), anti-pIRF3 (#4947), anti-phospho-NF-κB p65 (#3033) and anti-NF-κB p65 (#8242), purchased from Cell Signaling Technology; anti-His (ab18184), purchased from Abcam; anti-TRIM4 (#AP13289a), purchased from abcepta; anti-MDA5/IFIH1 (66770-1-Ig), purchased from Proteintech Group; anti-β-Actin (EM21002), anti-GFP (ET1607-31) and anti-GST (ET1611-47), purchased from HuaAn Biotechnology; Poly (dA: dT) naked (Invivogen, tlrl-patn-1), purchased from Neobioscience; Polyinosinic-polycytidylic acid potassium salt [poly (I:C)] (P9852, average MW: 200,000 to 500,000), purchased from Sigma-Aldrich.

### Plasmids

The following plasmids were used. Flag-nsp8 was kindly provided by the Pei-Hui Wang lab (Shandong University). HA-tagged Ub, K48-Ub (K48 only), and K63-Ub (K63 only) were kindly provided by Dr. Yang Du (Sun Yat-Sen University). To generate GFP-/Flag-/GST-tagged MDA5 or RIG-I and nsp8 or its mutants, the nsp8, MDA5 and RIG-I fragments were subcloned into the pEGFP-C2, pCMV-3Tag-1A and pEBG vectors, respectively. To generate Flag-, HA- or GST-tagged TRIM4 or nsp8 expressing plasmid, the TRIM4 or nsp8 fragments were subcloned into the pCMV-3Tag-1A, pcDNA3.1 or pEBG vectors, respectively.

### Transfection and luciferase reporter assays

HEK293T cells were seeded in 24-well plates overnight and then transfected using Lipofectamine 2000 (Invitrogen) with 100 ng ISRE luciferase reporter (firefly luciferase), 20 ng pRL-TK plasmid (Renilla luciferase), 150 ng Flag-MDA5 expressing plasmid and increasing amounts (0, 100, or 200 ng) of nsp8-expressing plasmid. Thirty-six hours post transfection, cells were collected, and luciferase activity was measured with a Dual-Luciferase Assay kit (Promega) with a Synergy2 Reader (Bio-Tek) according to the manufacturer’s protocol. The relative level of gene expression was determined by normalization of firefly luciferase activity to Renilla luciferase activity.

### Virus infection and viral RNA extraction

SARS-CoV-2 was provided by Dr. Ting Pan (School of Medicine, Sun Yat-Sen University), and infection experiments with SARS-CoV-2 were performed in the biological safety third-level laboratory of Zhongshan School of Medicine, Sun Yat-Sen University. HSV-1 stock and infection were performed as previously described [51]. Encephalomyocarditis Virus (EMCV) was provided by Dr. Ping Zhang (Department of Immunology, Zhongshan School of Medicine, Sun Yat-sen University), and amplified in Vero cells, then stored at -80°C. And VSV stock and infection were performed as previously described [52].

The SARS-CoV-2 replicon with deletion of the spike (S) gene was provided by Dr. Deyin Guo and described as previously [53]. The SARS-CoV-2 replicon BAC DNA was transiently transfected into cells to establish spike-defective SARS-CoV-2 infected cells in the biological safety second-level condition.

EMCV and VSV were amplified in Vero cells, the supernatants were collected and centrifuged at 8,000 × g for 15 min twice to remove cells and cell debris. Virions were purified and concentrated by dialysis in PEG8000-NaCl solution at 4°C overnight and then resuspended in PBS solution. Finally viral RNA was extracted using TRIzol (Invitrogen).

### shRNA

The following shRNA sequences were used, all sequences were cloned into pLKO.1 lentivirus expression vector. (1) RIG-I: 5’- AGCACTTGTGGACGCTTTAAA-3’;(2) MDA5: 5’-CCATCGTTTGAGAACGCTCAT-3’;(3) TRIM4: 5’-GAAGTTGAGAGTAGAGATA-3’; (4) RBBP6: 5’-CCTTTGATGGGCTCCACAT-3’;(5) HECTD1: 5’-GCACTTTCTTACCAGCCCTTT-3’; (6) HERC5: 5’-GAAGGACTAGACAATCAGAAA-3’; (7) TRIM25: 5’-CCGG AACAGTTAGTGGATTTA-3’;(8) TRIM65: 5’-GAATTATCGCAATCTGACCTT-3’;(9) TRIM13: 5’-GCTGTCTTTC AAACTTCACTT-3’; (9) TLR3: 5’-CCTCTTCGTAACTTGACCATT-3’.

### Real-time PCR

Total RNA was extracted using TRIzol reagent (Invitrogen) and subjected to reverse transcription using StarScript III All-in-one RT Mix (A230-10, GenStar). Real-time PCR was performed with 2×RealStar Green Fast Mixture (A301-10, GenStar). The following primers were used for real-time PCR.

1. *TNF*: F 5’-CTCTTCTGCCTGCTGCACTTTG-3’; R 5’-ATGGGCTACAGGCTTGTCACTC-3’
2. *IFNA*: F 5’-AGAAGGCTCCAGCCATCTCTGT-3’; R 5’-TGCTGGTAGAGTTCGGTGCAGA-3’
3. *ISG15*: F 5’-CTCTGAGCATCCTGGTGAGGAA-3’; R 5’-AAGGTCAGCCAGAACAGGTCGT-3’
4. *IFNB*: F 5’-CCTACAAAGAAGCAGCAA-3’; R 5’-TCCTCAGGGATGTCAAAG-3’
5. *IFIT1*: F 5’-GCCTTGCTGAAGTGTGGAGGAA-3’; R 5’-ATCCAGGCGATAGGCAGAGATC-3’
6. *IFIT2*: F 5’-GGAGCAGATTCTGAGGCTTTGC-3’; R 5’-GGATGAGGCTTCCAGACTCCAA-3’
7. *IL-6*: F 5’-AGACAGCCACTCACCTCTTCAG-3’; R 5’-TTCTGCCAGTGCCTCTTTGCTG-3’
8. *CCL20*: F 5’-AAGTTGTCTGTGTGCGCAAATCC-3’; R 5’-CCATTCCAGAAAAGCCACAGTTTT-3’
9. SARS-CoV-2 *N*: F 5’-CAATGCTGCAATCGTGCTAC-3’; R 5’-GTTGCGACTACGTGATGAGG-3’
10. SARS-CoV-2 *S*: F 5’-CAGATGCTGGCTTCATCAAA-3’; R 5’-GGTTGGCAATCAATTTTTGG-3’
11. *EMCV 3D*: F 5’-GTCATACTATCGTCCAGGGACTCTAT-3’; R 5’-CATCTGTACTCCACACTCTCGAATG-3’
12. *Actin*: F 5’-CACCATTGGCAATGAGCGGTTC-3’; R 5’-AGGTCTTTGCGGATGTCCACGT-3’
13. *IL-16*: F 5’-TTGGACACAGGGTTCTCGCTCA-3’; R 5’-AGCAGGGAGATAACGGACTGAC-3’
14. *IL-17F*: F 5’-AACCAGCGCGTTTCCATGTCAC-3’; R 5’-GAGCATTGATGCAGCCCAAGTTC-3’
15. *IL-17A*: F 5’-CGGACTGTGATGGTCAACCTGA-3’; R 5’-GCACTTTGCCTCCCAGATCACA-3’
16. *IL-33*: F 5’-GCCTGTCAACAGCAGTCTACTG-3’; R 5’-TGTGCTTAGAGAAGCAAGATACTC-3’
17. *GATA3*: F 5’-ACCACAACCACACTCTGGAGGA-3’; R 5’-TCGGTTTCTGGTCTGGATGCCT-3’
18. *NKG2B*: F 5’-AAAGTCGGCATCTCTGTGCTT-3’; R 5’-CGGTGTGCTCCTCACTGTA-3’
19. *NKP44*: F 5’-CTGAGTCTCCATCTACCATCCC-3’; R 5’-TCTTGGCTACGAGGAGTCCACA -3’
20. *TLR3*: F 5’-GTATTGCCTGGTTTGTTAATT-3’; R 5’-AAGAGTTCAAAGGGGGCACT-3’

### Immunoprecipitation and immunoblot analysis

For immunoprecipitation, whole cell extracts were prepared after transfection or stimulation with appropriate ligands, followed by incubation for 1 h at 4°C with anti-GFP/Flag/HA agarose beads (AlpaLife) or anti-GST agarose beads (Cytiva). Beads were washed 4 times with low-salt lysis buffer, and immunoprecipitants were eluted with 2x SDS loading buffer and then resolved by SDS-PAGE. Proteins were transferred to PVDF membranes (Millipore) and further incubated with the appropriate primary and secondary antibodies. The images were visualized using Odyssey Sa (LI-COR).

### Immunofluorescence staining

The cells were fixed with 4% formaldehyde in phosphate-buffered saline (PBS) for 30 min, permeabilized with methanol for 10 min, and blocked with 2% bovine serum albumin in PBS for 30 min. Then, the cells were incubated with primary antibody overnight at 4°C. After three washes with PBS containing 0.1% Triton X-100, the cells were incubated with Alexa Fluor 488- or 555-labelled anti-rabbit IgG antibodies (Invitrogen, A11034 and A27039) for 1 h at room temperature. The cells were counterstained with 4,6-diamidino-2-phenylindole (DAPI; Sigma-Aldrich, D9542) followed by three additional washes. The cells were mounted in antifade agent on glass slides and visualized with a confocal fluorescence microscope (Zeiss LSM800 microscopy).

### Pull down assays

HEK293T cells were transfected with pCMV-3Tag-1A-nsp8 using PEI Max (Sigma-Aldrich) and harvested 48h post-transfection. Cells were then lysed in NP40 buffer containing proteinase inhibitor cocktail (Roche) and Flag-nsp8 protein were purified with anti-Flag agarose beads (AlpaLife). GST-MDA5 or MDA5 CARDs was expressed in E. coli and purified using Glutathione agarose beads (Cytiva); His-TRIM4 or His-nsp8 was expressed in E. coli and purified using Ni-NTA beads (Cytiva). Glutathione agarose beads or Ni-NTA were pre-blocked with non-specific cell lysates and then incubated with the mixture of purified GST-MDA5 and/or His-TRIM4 in presence or absence of Flag-nsp8 proteins, respectively, for 2 h at 4°C. After extensive washing of the resin with buffer containing 0.1% NP40, the resin was analyzed by immunoblotting with indicated antibodies. For sequential double pull-down assays, the glutathione agarose beads were eluted with GSH solution (50 mM Tris-HCl, 400mM NaCl, 50 mM GSH, 1 mM DTT, 1 mM EDTA).

### In vitro and in vivo ubiquitination

For in vitro ubiquitination, GST-tagged MDA5 CARDs protein was purified using Glutathione agarose beads, His-tagged TRIM4 and nsp8 proteins were purified using nickel-agarose beads. The bead-bound MDA5 CARDs was re-suspended in buffer (50 mM Tris-HCl, pH 8.0, 5 mM MgCl2, 2 mM ATP, 0.5 mM DTT, 2 mM NaF) and followed with addition of ubiquitin and enzymes. The final concentrations were as follows: 200 ng/μL ubiquitin, 2 ng/μL E1, 20 ng/μL E2, and 200 ng/μL substrate in a total volume of 25 μL. The reactions were performed for 1 h at 37°C, then bead-bound proteins were washed three times with washing buffer (50 mM Tris-HCl, pH 8.0, 150 mM NaCl, 0.2% Triton X-100, 0.1% SDS) prior to analysis by western blotting. For in vivo ubiquitination, cells were transfected with GST-tagged MDA5 or MDA5 CARDs expressing plasmids together with a plasmid expressing HA-tagged K63-ubiquitin. Cells were harvested and lysed in 2% SDS in TBS (10 mM Tris-HCl, pH 8.0) at 95°C for 10 min. The lysates were diluted 20-fold with TBS containing 0.2% Triton X-100 and 2 mM EDTA to final SDS concentration of 0.1%, then incubated on a shaker at 4°C for 30 min. The lysates were incubated for 2 h at 4°C with anti-GST agarose beads, and then beads were washed three times with TBS containing 0.2% Triton X-100 and 0.1% SDS. Beads-binding proteins were resolved by SDS-PAGE, transferred, and immunoblotted with the indicated antibodies.

### Enzyme-linked immunosorbent assay (ELISA)

The supernatants were collected and the secretion of IFNβ and TNFα were measured using IFNβ and TNFα ELISA kits according to the standard manufacturer’s protocols (EHC026b and EHC103a, NeoBioscience Technology Co., Ltd., Shenzhen, China).

### Computer-based prediction and structural modeling

nsp8.pdb, MDA5 CARDs.pdb, K63-Ub.pdb and RIG-I CARDs.pdb were generated in SWISS-MODEL [54]. MDA5 CARDs.pdb or RIG-I CARDs.pdb was input into ZDOCK-SERVER [55] as a receptor, while nsp8 or K63-Ub was input as a ligand for docking computation. MDA5 CARDs or RIG-I CARDs.pdb with nsp8.pdb and MDA5 CARDs or RIG-I CARDs.pdb with K63-Ub.pdb were the best fit prediction models chosen from the results. All the pdb files were processed and visualized with PyMOL (Schrödinger).

### Statistical analyses

All statistical analysis was performed in Prism 7 software (GraphPad Software, La Jolla, CA, USA). One-way or Two-way ANOVA by Sidak’s multiple comparisons test, and multiple t-tests were performed. Statistical significance was defined as p value<0.05.

## Supporting information

S1 Fig Validation of SARS-CoV-2 nonstructural proteins in the regulation of expression of cytokines and inflammatory factors. A. HEK293T cells were transfected with empty vector (Ev) or Flag-NSP expressing plasmids. Twenty-four hours post transfection, cells were treated with poly(I:C) (5 ug/ml) for the indicated time, and total RNA was subjected to RT-PCR analysis of *TNF*, *IFNB*, *IFIT1*, *IFIT2*, *IL-6* and *CCL20* expression. The results are shown as the mean ± SD (n = 3), *, p < 0.05; **, p < 0.01; ***, p < 0.001; ****, p < 0.0001, by Sidak’s multiple comparisons test. B. The expression levels of individual viral protein were shown for Fig 1A. C. The expression of nsp8 was shown for Fig 1C.
(TIF)

S2 Fig Nsp8 suppresses MDA5-associated immune responses. A. HEK293T cells were transfected with an empty vector or increasing amounts of nsp8-expressing plasmid together with RIG-I, MDA5, MAVS, TBK1 or IRF3-expressing plasmid respectively. 36 h post transfection, cell lysates were subjected immunoblotting analysis with the indicated antibodies. B. HEK293T cells were transfected with an empty vector or nsp8-expressing plasmid plus an ISRE-luc reporter along with cGAS- and STING- expressing plasmids. 36 h post transfection, the cells were collected, and then cell lysates were analyzed for ISRE-luc activity. C. HEK293T cells were co-transfected with scramble or TLR3 shRNA, vector or nsp8-expressing plasmids, and ISRE-firefly luciferase reporter with renilla luciferase as internal control, and then untreated or treated with poly(I:C) (5 ug/ml) for 18 h. Then the cells were collected and the luciferase-based ISRE activity in cell lysates were measured (left), the total RNA were extracted and reverse-transcribed and detected by real-time PCR analysis (right). The results are shown as the mean ± SD (n = 3), ****, p < 0.0001, by Sidak’s multiple comparisons test. D. HEK293T cells were transfected with empty vector or Flag-nsp8. 48 h post transfection, cells were subjected to MTS assay. E-F. A549 cells were transfected with empty vector (Ev) or Flag-nsp8 plasmids. 24 h post transfection, cells were stimulated with poly(dA:dT) (5 ug/ml) (E) or infected with HSV-1 (MOI = 1) (F) for 18 h, and total RNA was subjected to RT-PCR analysis for *TNF* and *IFNB* expression. G. The knockdown efficiency of MDA5 and RIG-I in HEK293T cells were analyzed by immunoblotting analysis.
(TIF)

S3 Fig Predicted structure and validation of Nsp8 interaction with MDA5 CARDs but not RIG-I CARDs. A. The PDB structure of nsp8 was processed in PyMOL, the cartoon structure and surface structure are demonstrated. B. The predicated nsp8 structure was similar to the nsp8 structure in viral replication complex. The nsp8 structure was filtered from the structure of replicating SARS-CoV-2 polymerase (PDB: 6YYT) and compared with the predicated structure. C. PDB structures were input into the ZDOCK Server separately for docking calculations. The predicted binding models of MDA5 CARDs with nsp8 and MDA5 CARDs with K63-Ub were processed in PyMOL for demonstration. Model alpha simulates the protein surface. Red chain, nsp8; violet chain, MDA5 CARDs; brown chain, K63-Ub. D. Model alpha in (C) was subjected to vacuum electrostatics calculation in PyMOL. A3 and A4 indicate the viewing angle in the green frame. The green frame indicates the contact area demonstrated in A3 and A4. The scale bar indicates the range of vacuum electrostatics. E. HEK293T cells were transfected with an empty vector or nsp8-expressing plasmid or nsp8 point-mutated mutants plus an ISRE-luc reporter. 24 h post transfection, cells were treated with poly(I:C) (5 μg/ml) for 24 h and then the cells were collected, the cell lysates were analyzed for ISRE-luc activity. The results are shown as the mean ± SD (n = 3), ****, p < 0.0001, by Sidak’s multiple comparisons test. F. PDB structures were input into ZDOCK Server for docking calculation separately. The predicted binding models of RIG-I CARDs with nsp8 and RIG-I CARDs with K63-Ub were processed in PyMOL for demonstration. Model gamma simulates the protein surface. Red chain, nsp8; violet chain, RIG-I CARDs; brown chain, K63-Ub. G. Model gamma in (F) was subjected to vacuum electrostatics calculation in PyMOL. A5 and A6 indicate the viewing angle in the green frame. The green frame indicates the contact area demonstrated in A5 and A6. Scale bar indicates the range of vacuum electrostatics. H. Polar contacts within interface of RIG-I CARDs-nsp8 were demonstrated with PyMOL. B3, B4 indicates the viewing angle of dashed borders. #1, 2 indicates paired residues. Paired residues were highlighted with sticks model in yellow color. Green dashed lines indicate polar contacts between paired residues, number besides dashed line indicates distance between two atoms connected (Å).
(TIF)

S4 Fig Nsp8 but not Nsp2 or Nsp7 inhibits K63-linked MDA5 polyubiquitination. A. HEK293T cells were co-transfected with HA-K63-Ub and GST-MDA5 or GST-tagged empty vector plus Flag-nsp2 or Flag-nsp7 or Flag-nsp8. Twenty hours post transfection, cells were treated with MG132 (10 μM) for 4 h, and then cells were collected and lysed in 0.1% SDS-containing lysis buffer. Cell lysates were subjected to coimmunoprecipitation using anti-GST beads, followed by immunoblotting analysis with the indicated antibodies. B. The expression levels of endogenous MDA5, HA-K63-Ub and nsp8 were shown for Fig 4F.
(TIF)

S5 Fig Identification of TRIM4 as target of Nsp8 inhibition in MDA5-mediated antiviral responses and functional sites in MDA5 CARDs. A-B. HEK293T cells were co-transfected with scramble or specific shRNA of 7 E3 ligases individually as indicated, vector or nsp8-expressing plasmids, and ISRE-firefly luciferase reporter with renilla luciferase as internal control, and then cells were collected and the luciferase-based ISRE activity in cell lysates were measured (A); the total RNA were extracted and reverse-transcribed and detected by real-time PCR analysis (B). The results are shown as the mean ± SD (n = 3), *, p < 0.05; **, p < 0.01; ***, p < 0.001; ****, p < 0.0001, by Sidak’s multiple comparisons test. C-D. HEK293T cells were co-transfected with Flag-nsp8, GFP-MDA5 and HA-TRIM4-expressing plasmids as indicated. 36 h post transfection, the cells were collected and lysed, and then subjected to coimmunoprecipitation with anti-GFP beads (C) or anti-Flag beads (D), followed with immunoblots as indicated. E. Control (WT) and MDA5 knockdown (shMDA5) HEK293T cells were co-transfected with Flag-nsp8 and HA-TRIM4-expressing plasmids. 36 h post transfection, the cells were collected and lysed, and then subjected to coimmunoprecipitation with anti-HA beads, followed with immunoblots as indicated. F. HEK293T cells were co-transfected with GFP-MDA5 and HA-TRIM4-expressing plasmids, and then left untreated or infected with EMCV (MOI = 0.25) for 18 hours. And then coimmunoprecipitation with anti-GFP beads and immunoblotting analysis were performed as indicated. G. GST-TRIM4 and Flag-nsp8-expressing plasmids were co-transfected into HEK293T cells with HA-Ub expressing plasmid, infected with EMCV (MOI = 0.25) for 18 h. The cells were collected, lysed and subjected to ubiquitination assay with immunoprecipitation with anti-GST beads and western blotting with anti-HA antibody as indicated.
(TIF)

S6 Fig Nsp8 slightly downregulated MDA5 expression. The expression levels of endogenous MDA5 and nsp8 were shown for Fig 8B.
(TIF)
