# Supplementary figures and images for: SARS-CoV-2 Nsp8 suppresses MDA5 antiviral immune responses by impairing TRIM4-mediated K63-linked polyubiquitination
Source: PLoS Pathog. 2023 Nov 13;19(11):e1011792. doi: 10.1371/journal.ppat.1011792 (PMC10681309; doi:10.1371/journal.ppat.1011792)

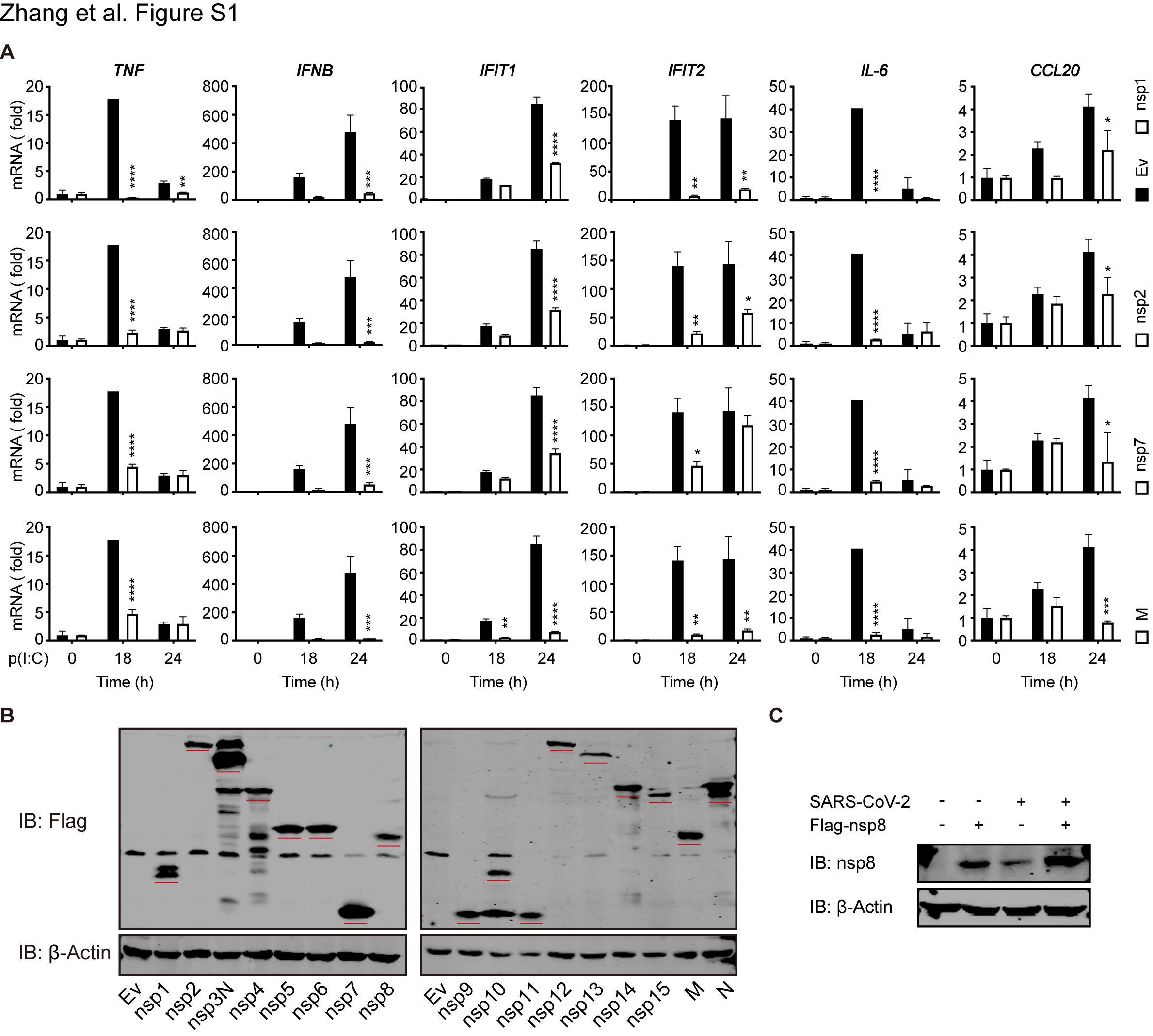

Supplement: S1 Fig — A. HEK293T cells were transfected with empty vector (Ev) or Flag-NSP expressing plasmids. Twenty-four hours post transfection, cells were treated with poly(I:C) (5 ug/ml) for the indicated time, and total RNA was subjected to RT-PCR analysis of TNF, IFNB, IFIT1, IFIT2, IL-6 and CCL20 expression. The results are shown as the mean ± SD (n = 3), *, p < 0.05; **, p < 0.01; ***, p < 0.001; ****, p < 0.0001, by Sidak’s multiple comparisons test. B. The expression levels of individual viral protein were shown for Fig 1A. C. The expression of nsp8 was shown for Fig 1C. (TIF) [file ppat.1011792.s001.tif]

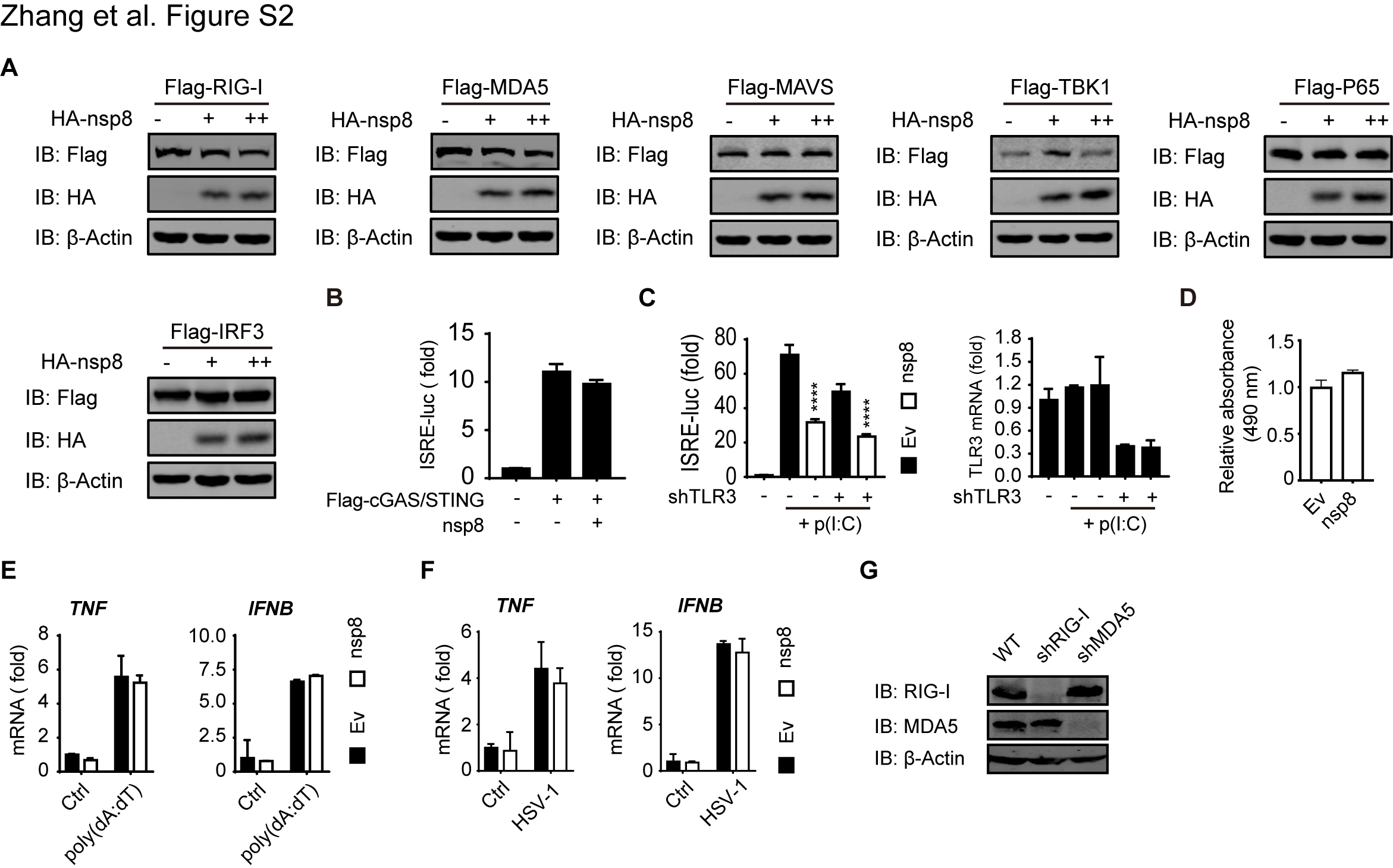

Supplement: S2 Fig — A. HEK293T cells were transfected with an empty vector or increasing amounts of nsp8-expressing plasmid together with RIG-I, MDA5, MAVS, TBK1 or IRF3-expressing plasmid respectively. 36 h post transfection, cell lysates were subjected immunoblotting analysis with the indicated antibodies. B. HEK293T cells were transfected with an empty vector or nsp8-expressing plasmid plus an ISRE-luc reporter along with cGAS- and STING- expressing plasmids. 36 h post transfection, the cells were collected, and then cell lysates were analyzed for ISRE-luc activity. C. HEK293T cells were co-transfected with scramble or TLR3 shRNA, vector or nsp8-expressing plasmids, and ISRE-firefly luciferase reporter with renilla luciferase as internal control, and then untreated or treated with poly(I:C) (5 ug/ml) for 18 h. Then the cells were collected and the luciferase-based ISRE activity in cell lysates were measured (left), the total RNA were extracted and reverse-transcribed and detected by real-time PCR analysis (right). The results are shown as the mean ± SD (n = 3), ****, p < 0.0001, by Sidak’s multiple comparisons test. D. HEK293T cells were transfected with empty vector or Flag-nsp8. 48 h post transfection, cells were subjected to MTS assay. E-F. A549 cells were transfected with empty vector (Ev) or Flag-nsp8 plasmids. 24 h post transfection, cells were stimulated with poly(dA:dT) (5 ug/ml) (E) or infected with HSV-1 (MOI = 1) (F) for 18 h, and total RNA was subjected to RT-PCR analysis for TNF and IFNB expression. G. The knockdown efficiency of MDA5 and RIG-I in HEK293T cells were analyzed by immunoblotting analysis. (TIF) [file ppat.1011792.s002.tif]

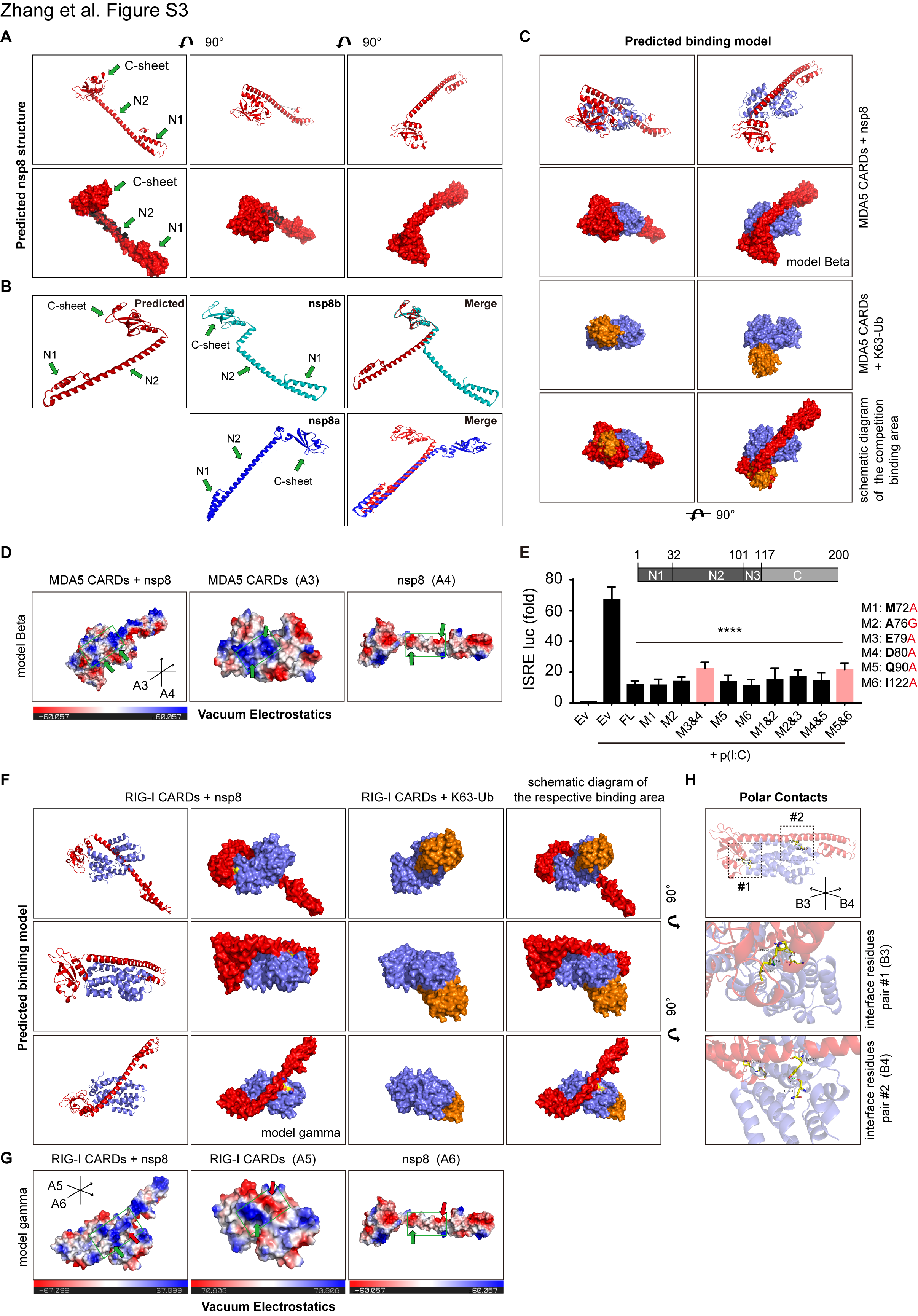

Supplement: S3 Fig — A. The PDB structure of nsp8 was processed in PyMOL, the cartoon structure and surface structure are demonstrated. B. The predicated nsp8 structure was similar to the nsp8 structure in viral replication complex. The nsp8 structure was filtered from the structure of replicating SARS-CoV-2 polymerase (PDB: 6YYT) and compared with the predicated structure. C. PDB structures were input into the ZDOCK Server separately for docking calculations. The predicted binding models of MDA5 CARDs with nsp8 and MDA5 CARDs with K63-Ub were processed in PyMOL for demonstration. Model alpha simulates the protein surface. Red chain, nsp8; violet chain, MDA5 CARDs; brown chain, K63-Ub. D. Model alpha in (C) was subjected to vacuum electrostatics calculation in PyMOL. A3 and A4 indicate the viewing angle in the green frame. The green frame indicates the contact area demonstrated in A3 and A4. The scale bar indicates the range of vacuum electrostatics. E. HEK293T cells were transfected with an empty vector or nsp8-expressing plasmid or nsp8 point-mutated mutants plus an ISRE-luc reporter. 24 h post transfection, cells were treated with poly(I:C) (5 μg/ml) for 24 h and then the cells were collected, the cell lysates were analyzed for ISRE-luc activity. The results are shown as the mean ± SD (n = 3), ****, p < 0.0001, by Sidak’s multiple comparisons test. F. PDB structures were input into ZDOCK Server for docking calculation separately. The predicted binding models of RIG-I CARDs with nsp8 and RIG-I CARDs with K63-Ub were processed in PyMOL for demonstration. Model gamma simulates the protein surface. Red chain, nsp8; violet chain, RIG-I CARDs; brown chain, K63-Ub. G. Model gamma in (F) was subjected to vacuum electrostatics calculation in PyMOL. A5 and A6 indicate the viewing angle in the green frame. The green frame indicates the contact area demonstrated in A5 and A6. Scale bar indicates the range of vacuum electrostatics. H. Polar contacts within interface of RIG-I CARDs-nsp8 were demon [file ppat.1011792.s003.tif]

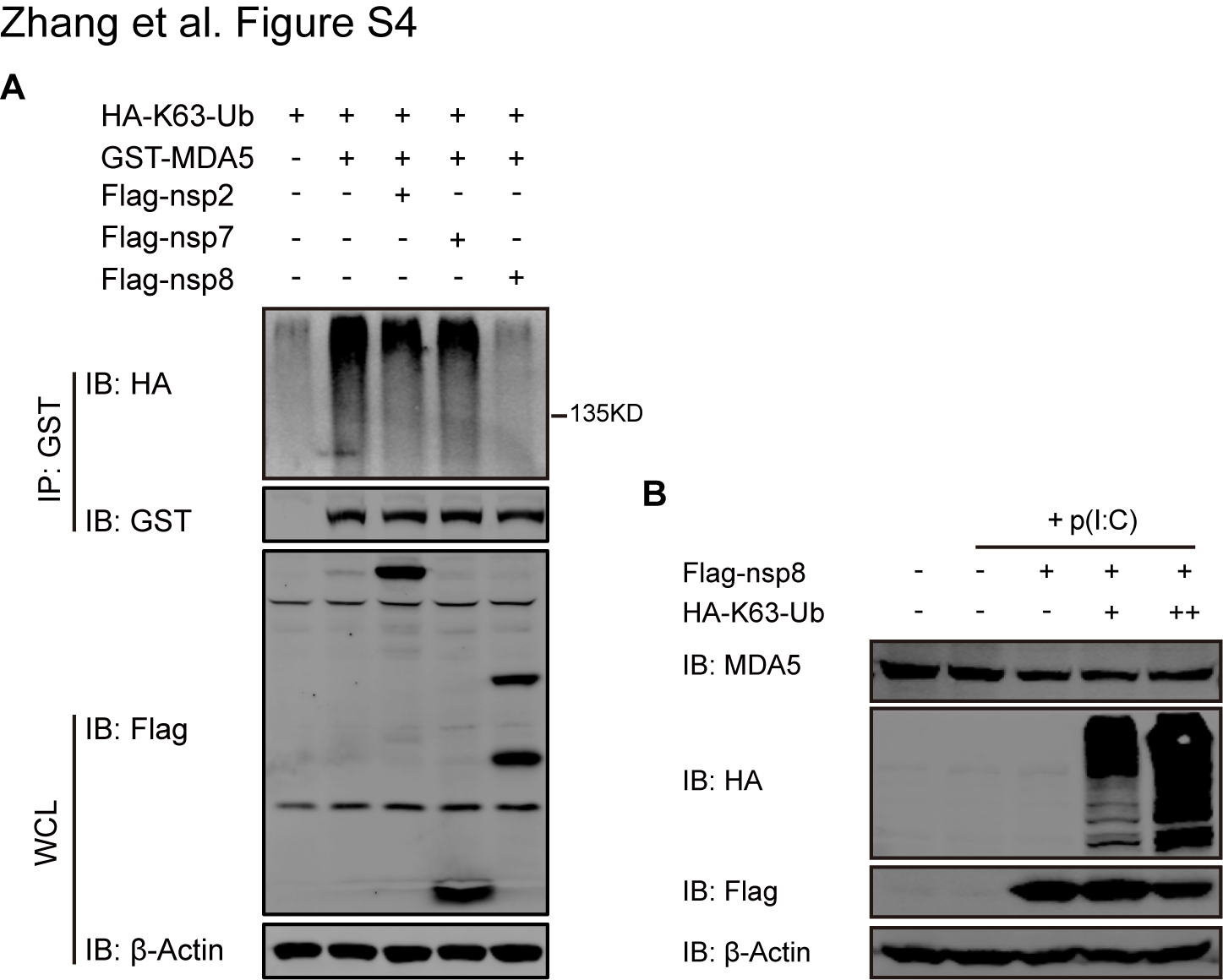

Supplement: S4 Fig — A. HEK293T cells were co-transfected with HA-K63-Ub and GST-MDA5 or GST-tagged empty vector plus Flag-nsp2 or Flag-nsp7 or Flag-nsp8. Twenty hours post transfection, cells were treated with MG132 (10 μM) for 4 h, and then cells were collected and lysed in 0.1% SDS-containing lysis buffer. Cell lysates were subjected to coimmunoprecipitation using anti-GST beads, followed by immunoblotting analysis with the indicated antibodies. B. The expression levels of endogenous MDA5, HA-K63-Ub and nsp8 were shown for Fig 4F. (TIF) [file ppat.1011792.s004.tif]

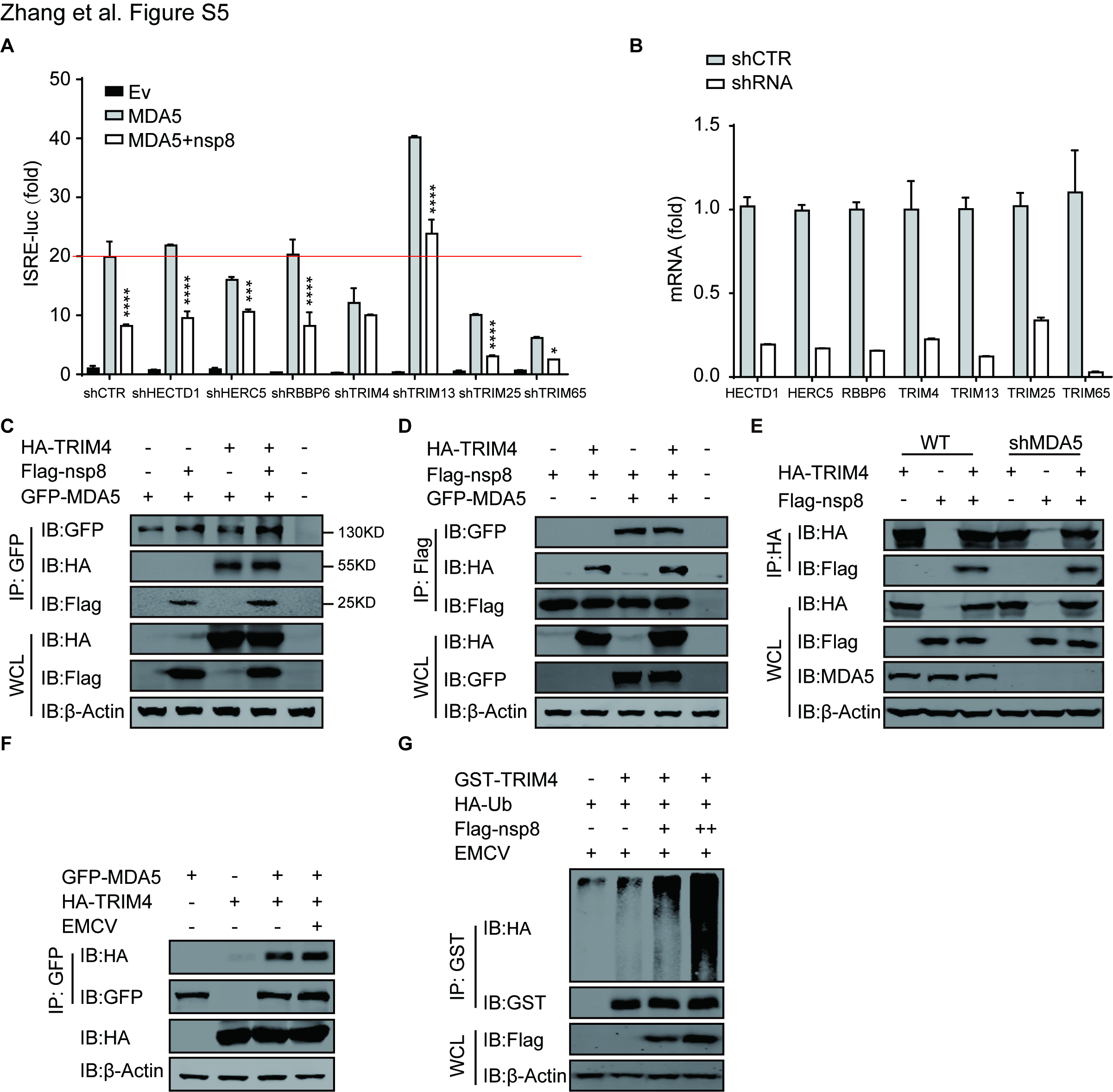

Supplement: S5 Fig — A-B. HEK293T cells were co-transfected with scramble or specific shRNA of 7 E3 ligases individually as indicated, vector or nsp8-expressing plasmids, and ISRE-firefly luciferase reporter with renilla luciferase as internal control, and then cells were collected and the luciferase-based ISRE activity in cell lysates were measured (A); the total RNA were extracted and reverse-transcribed and detected by real-time PCR analysis (B). The results are shown as the mean ± SD (n = 3), *, p < 0.05; **, p < 0.01; ***, p < 0.001; ****, p < 0.0001, by Sidak’s multiple comparisons test. C-D. HEK293T cells were co-transfected with Flag-nsp8, GFP-MDA5 and HA-TRIM4-expressing plasmids as indicated. 36 h post transfection, the cells were collected and lysed, and then subjected to coimmunoprecipitation with anti-GFP beads (C) or anti-Flag beads (D), followed with immunoblots as indicated. E. Control (WT) and MDA5 knockdown (shMDA5) HEK293T cells were co-transfected with Flag-nsp8 and HA-TRIM4-expressing plasmids. 36 h post transfection, the cells were collected and lysed, and then subjected to coimmunoprecipitation with anti-HA beads, followed with immunoblots as indicated. F. HEK293T cells were co-transfected with GFP-MDA5 and HA-TRIM4-expressing plasmids, and then left untreated or infected with EMCV (MOI = 0.25) for 18 hours. And then coimmunoprecipitation with anti-GFP beads and immunoblotting analysis were performed as indicated. G. GST-TRIM4 and Flag-nsp8-expressing plasmids were co-transfected into HEK293T cells with HA-Ub expressing plasmid, infected with EMCV (MOI = 0.25) for 18 h. The cells were collected, lysed and subjected to ubiquitination assay with immunoprecipitation with anti-GST beads and western blotting with anti-HA antibody as indicated. (TIF) [file ppat.1011792.s005.tif]

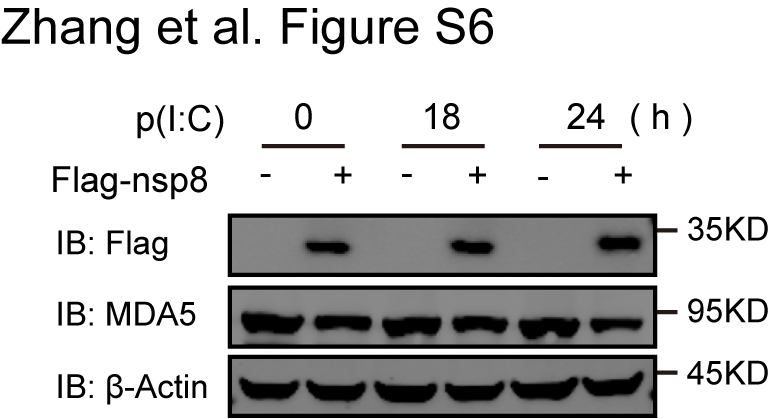

Supplement: S6 Fig — The expression levels of endogenous MDA5 and nsp8 were shown for Fig 8B. (TIF) [file ppat.1011792.s006.tif]
